# Potential changes in the connectivity of marine protected areas driven by extreme ocean warming

**DOI:** 10.1038/s41598-021-89192-6

**Published:** 2021-05-14

**Authors:** Luciana Shigihara Lima, Douglas Francisco Marcolino Gherardi, Luciano Ponzi Pezzi, Leilane Gonçalves dos Passos, Clarissa Akemi Kajiya Endo, Juan Pablo Quimbayo

**Affiliations:** 1 Laboratory of Ocean and Atmosphere Studies (LOA), Earth Observation and Geoinformatics Division, National Institute for Space Research (INPE), São José dos Campos, SP Brazil; 2 Geophysical Institute and Bjerknes Centre for Climate Research, University of Bergen, P.O. Box 7803, 5020 Bergen, Norway; 3 Nansen Environmental and Remote Sensing Center, Bergen, Norway; 4 Institute of Marine Research, P.O. Box 1870 Nordnes, 5817 Bergen, Norway; 5 Centre for Ecological and Evolutionary Synthesis (CEES), Department of Biosciences, University of Oslo, P.O. Box 1066 Blindern, 0316 Oslo, Norway; 6 Centro de Biologia Marinha (CEBIMar), Universidade de São Paulo, São Sebastião, 11612-109 Brazil

**Keywords:** Ocean sciences, Climate-change ecology, Ecological modelling

## Abstract

Projected future climate scenarios anticipate a warmer tropical ocean and changes in surface currents that will likely influence the survival of marine organisms and the connectivity of marine protected areas (MPAs) networks. We simulated the regional effects of climate change on the demographic connectivity of parrotfishes in nine MPAs in the South Atlantic through downscaling of the HadGEM2-ES Earth System Model running the RCP 8.5 greenhouse gas trajectory. Results indicate a tropicalization scenario over the tropical southwest Atlantic following an increase of sea surface temperature (SST) between 1.8 and 4.5 °C and changes in mean surface currents between − 0.6 to 0.5 m s^−1^ relative to present conditions. High mortality rates will reduce demographic connectivity and increase the isolation of oceanic islands. The simulation of organismal response to ocean warming shows that acclimation can significantly improve (*p* < 0.001) particle survival, promoting connectivity and tropicalization of MPAs, with potential impacts on their functional integrity and long-term resilience.

## Introduction

Marine protected areas (MPAs) are important elements of conservation policy that aim to promote connectivity between the populations they support (hereafter referred to as MPA connectivity) and to maintain trophic webs under disturbance regimes^1–3^. Herbivorous reef fishes, such as parrotfishes, can be a key component in this process, since they are responsible for modulating the composition and standing biomass of algal assemblages, and for helping in the replenishment and potential recovery of coral populations^2, 4–6^. Individuals of the *Sparisoma* genus found in the southwestern tropical Atlantic have a diet based primarily on detritus and macroalgae, representing an important group responsible for the transfer of nutrients and energy from primary producers to higher trophic levels^7^. Several tropical reef fishes live in the limit of their thermal tolerance and can travel long distances, ocean warming can result in an expansion or shift of larval dispersal into higher latitudes^8^. Changes in this sense can lead to disturbances in local ecological network structures and disruption of the functional integrity of reef ecosystems^9, 10^. For example, parrotfishes are ectotherms, and their rates of biochemical and cellular processes are governed mainly by environmental temperature. Therefore, large positive anomalies in ocean temperature will intensify their metabolic demand and change both their geographical distribution, as well as trophic interactions^8^. Considering the climate warming over the tropics above the current biological tolerance, individuals capable of changing their physiological limits across generations through acclimation (including parrotfishes) will also influence how reef fishes respond with shifts in environmental conditions and spatial distribution^10, 11^. In this sense, some studies have proposed that species with wide geographic distribution will have higher potential for acclimation than species with narrow distribution, since they probably have higher plasticity in thermal tolerance^12, 13^. For instance, tropical wrasses that live in small tropical areas exhibited mitochondrial and cardiac failure with elevated temperature and therefore may be most vulnerable to the impact of rising sea surface temperature (SST)^14^. Conversely, those species that exhibited thermal acclimation are less vulnerable but can have high energetic cost and variation in their life-history traits that influence negatively the maintenance of their populations. To our knowledge, there are no published records on the capacity of parrotfish to acclimate as a response to ocean warming but there is growing evidence that this may occur in tropical reef fishes both as non-genetic parental effects or as genetically induced reproductive plasticity^13, 15^.

The Representative Concentration Pathway (RCP) are different scenarios for greenhouse gas concentration proposed by the Intergovernmental Panel on Climate Change (IPCC). The RCP provides different representations of climate considering radiative forcing^16^. The RCP 8.5, an extreme warming scenario, projects that 81% of terrestrial and 37% of marine assemblages will have at least one species exposed to unprecedented high mean annual temperatures before 2100, and the percentages can be even more significant in the tropics^17^. Despite the controversy around the likelihood of this so-called business-as-usual emission trajectory^18^, the RCP 8.5 can be used as a worst-case scenario for which important outcomes, and their seasonal and interannual variability, should be considered in exploratory research for a high-risk future for the ecosystem. Using the RCP 8.5 scenario also helps to compare systems response globally as it has been the most used in studies trying to predict ecological impacts in marine ecosystems under climate change^19^. The projected global mean increase in SST around 0.035 °C year^-1^ will cause the community safety margin in tropical MPAs, defined as the inherent buffer against warming based on the thermal sensitivity of the biologic community, to be exceeded by 2050^9^. As a result, adverse impacts are expected for tropical species living close to their maximum thermal tolerance, especially in isolated marine reserves such as those in oceanic islands of the south Atlantic^20^.

The Target eleven of the Convention on Biological Diversity highlights the importance of MPAs, and recommends the increase in open ocean protected sites that are ecologically representative and spatially integrated into more extensive networks^21^. The establishment of broader seascape networks can ensure proper ecological and demographic connectivity as well as adaptation to climate change^22^. Therefore, the integration of MPAs through demographic connectivity in the future needs to be assessed, considering different organisms responses to ocean warming. Furthermore, it should include mechanisms involving thermal tolerance and adaptation of individuals and changes in the atmosphere and surface ocean dynamics^12, 22^. For instance, warmer waters may not only increase the mortality of individuals but also accelerate the development of fish larvae, causing a reduction in pelagic larval duration (PLD)^23–25^. It will also directly affect the productivity capacity and energy flow within food webs^26^. Shifts in the timing and output of reproduction, premature reef-seeking larval behaviour, increase in self-recruitment and reduction in the magnitude of demographic connectivity are also expected to occur^27, 28^. Another significant consequence of ocean warming is the redistribution of species caused by increased colonization of warm-adapted species in subtropical sites^29, 30^. Alterations in biological behaviour are especially evident in transition zones, where both tropical and temperate species overlap and changes in SST are more pronounced^8^. Empirical studies have shown that the arrival of typical tropical herbivorous fishes into subtropical sites promotes new trophic interactions and alters the structure of kelp forests, resulting in a process known as tropicalization^8^.

The influence of ocean warming on MPA connectivity should also consider the fact that some organisms will be able to increase their thermal tolerance and maintain growth and swimming ability by epigenetic inheritance. Experiments with a tropical reef fish involving the rearing of siblings of the damselfish *Acanthochromis polyacanthus* have shown that it is capable of transgenerational metabolic acclimation (including egg clutches) and it can adapt to an increase of up to + 3 °C in SST^13, 15, 31^. This has been taken as evidence that acclimation of tropical reef fishes may happen in time scales shorter than the rate of ocean water warming induced by anthropogenic climate change. Nevertheless, assuming that thermal preference can drive fish behaviour before ocean temperature raises, reef fishes may migrate to colder areas before they develop acclimation^32, 33^.

In this study, we combine a dynamical downscaling using an eddy resolving ocean model and a individual based model set with the biological characteristics of the widely distributed reef fish genus *Sparisoma* spp. to investigate the potential impacts of future ocean warming on the demographic connectivity of MPAs in the southwestern tropical Atlantic. This reef fish group represents an important component of tropical reefs, due to their functional roles in bioerosion, sediment production and biomass control of macroalgae^6^. We complement our analysis determining the connectivity of MPAs for an alternative reef fish acclimation scenario. Here, we took recent evidences of transgenerational acclimation found in a tropical reef fish (*Acanthochromis polyacanthus*) as indication of the acclimation potential of *Sparisoma* eggs and larvae. We assume that this mechanism can be reasonably generalised to other reef fishes and their propagules considering that even small changes in water temperature (+ 1.5 °C) can directly influence the thermal tolerance of offsprings^13, 15^.

## Results

### Projected changes over the tropical Atlantic

**Figure 1:**
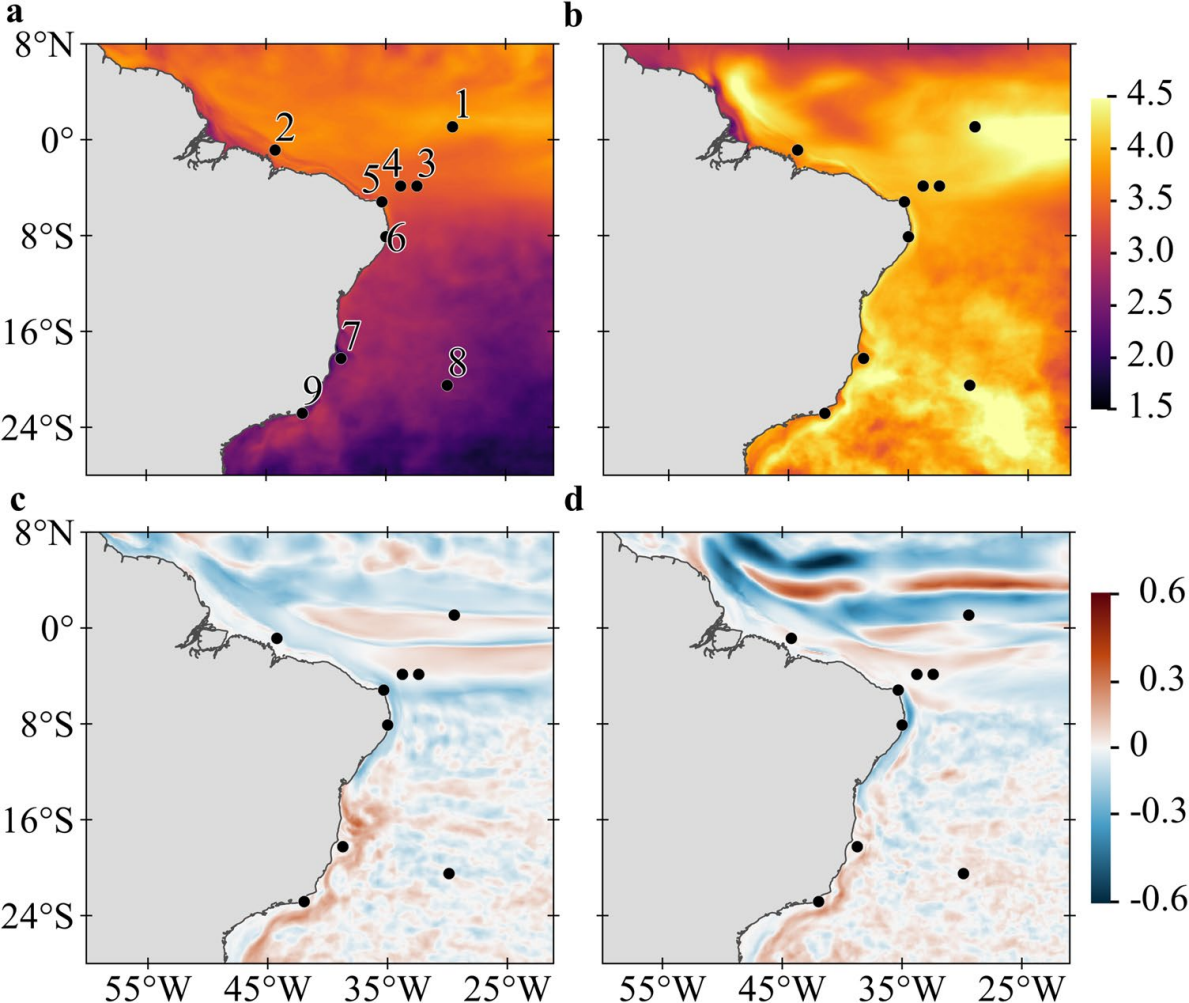
Differences between future (RCP8.5, 2092–2099) and historic (1997–2004) simulations for SST and current magnitude averaged for the ocean upper 100 m layer. (**a**) Summer (JFM) and (**b**) Winter (JAS) for SST (°C); larger differences of temperature between simulations are shown in yellow tones and smaller differences in purple tones. (**c**) Summer (JFM) and (**d**) Winter (JAS) for current magnitude (m s^−1^); red tones indicate current strengthening and blue tones indicate current weakening. Black dots represent MPA locations: (1) São Pedro e São Paulo Archipelago (SPSP); (2) Manuel Luís (ML); (3) Fernando de Noronha Archipelago (FN); (4) Atol das Rocas (AR); (5) Recifes de Corais (RC); (6) Costa dos Corais (CC); (7) Abrolhos (AB); (8) Martim Vaz e Trindade Archipelago (TR); and (9) Arraial do Cabo and Cabo Frio region (CF). Created with Matlab R2019a (www.mathworks.com) and QGIS-3.4 (https://www.qgis.org).

The study region covers most of the Southwestern Atlantic, including the Brazilian coast and four oceanic islands, representing all federal MPAs with reefs in the tropical Brazilian exclusive economic zone (Fig. 1). Parrotfish spawning and recruitment were simulated to compute the connectivity of five sites on the continental shelf: Manuel Luís (ML), Recifes de Corais (RC), Costa dos Corais (CC), Abrolhos (AB) and Arraial do Cabo and Cabo Frio region (CF) and four oceanic islands: Atol das Rocas (AR), Fernando de Noronha Archipelago (FN), São Pedro e São Paulo Archipelago (SPSP) and Martim Vaz e Trindade Archipelago (TR) (details in Supplementary Fig. 1). We sought to identify potential changes in ocean temperature and current velocity in these sites that could influence survival rates and alter dispersion dynamics^28, 29, 34^, leading to new spatial arrangement of habitats^35^. The impacts of these potential changes were evaluated in two different biophysical simulations, a non-acclimated scenario assuming present-day thermal tolerance of *Sparisoma* (24 to 30 °C) and an acclimated scenario where eggs and larvae are able to adapt to an increase of up to + 3 °C in SST^13, 15, 31^.

Based on this premise, we compared present and future ocean states by dynamically downscaling the worst-case (extreme) RCP 8.5 ocean warming scenario produced by the Earth System Model (ESM) HadGEM2-ES, available from the Coupled Model Intercomparison Project 5 (CMIP5). Ocean downscaling was computed using the Regional Ocean Model System (ROMS, version 3.7)^36^ for the period of 1997–2005 and 2092–2100, representing historical and extreme warming scenario, respectively. The dynamical downscaling has the objective of producing regional simulations with a high horizontal resolution (approximately 9.3 km) forced with a low-resolution ESM (208 km × 139 km at the equator). This allows the ocean model to resolve mesoscale processes such as eddies, that are important for the dispersal and recruitment of fish eggs and larvae^37, 38^. The atmospheric forcing and ocean boundary conditions used in both simulations come from the HadGEM2-ES model^39^, ensemble r2i1p1 (Supplementary Table 1), and included river discharge. The inherent uncertainties involved in using low-resolution ESM data to produce high-resolution local information were assessed by downscaling the historical scenario and comparing the results with observations. This configuration allows for an indirect accuracy assessment of model downscaling of future extreme ocean warming. For that purpose, the historical simulation was compared with satellite SST from the Operational Sea Surface Temperature and Sea Ice Analysis (OSTIA)^40^ and surface currents from the Simple Ocean Data Assimilation (SODA, version 3.4.2)^41^.

**Table 1:** Areas of each MPA used in this study, which the number of released particles launched from each, and the coordinates of the centroid in each location.

MPA	Area (km^2^)	Number of released particles	Coordinates	Number of cells in grid model
SPSP	125.6	2133	0°54′N, 29°18′W	4
ML	1257.6	8530	0°55′N, 44°20′W	16
FN	447.2	4247	3°51′S, 32°29′W	8
AR	308.7	2123	3°51′S, 33°48′W	4
RC	1507.4	7932	5°13′S, 35°18′W	17
CC	3995.4	21,298	9°12′S, 35°13′W	48
AB	992.4	4824	17°59′S, 38°39′W	10
TR	2299.9	11,221	20°30′S, 29°04′W	24
CF	1616.6	7692	22°53′S, 41°6′W	23

SPSP, São Pedro and São Paulo Archipelago; ML, Manuel Luís Parcel; FN, Fernando de Noronha Archipelago; AR, Atol das Rocas; RC, Recife de Corais; CC, Costa de Corais; AB, Abrolhos; TR, Trindade and Martim Vaz Archipelago; CF, Cabo Frio.

The impacts of climate change on the seasonal SSTs of the tropical southwest Atlantic point to an overall surface warming (relative to the historical scenario) between 1.8 and 4.5 °C in most of the equatorial region (Fig. 1a,b). This warming will be lower during the austral summer in open ocean regions south of 5° S and over the shelf south of 15° S (Fig. 1a). These values contrast with more widespread warming ranging from 3.5 to 4.5 °C during the austral winter with higher values at the equator and along with the core of the Brazil Current (BC, see Supplementary Fig. 2 for currents position) offshore the continental shelf (Fig. 1b). Our simulations agree with previous studies which suggest that the BC is intensifying and shifting southwards due to a poleward shift of near-surface ocean wind^42, 43^. The dynamical downscaling also points to marked changes in surface current velocities in the extreme ocean warming scenario with intense surface flow changes occurring between 5°S and 8°N (Fig. 1c,d). The NBC retroflection is expected to become weaker (− 0.6 m s^−1^) than today and the northern branch of the South Equatorial Current (nSEC) stronger (0.5 m s^−1^).

**Figure 2:**
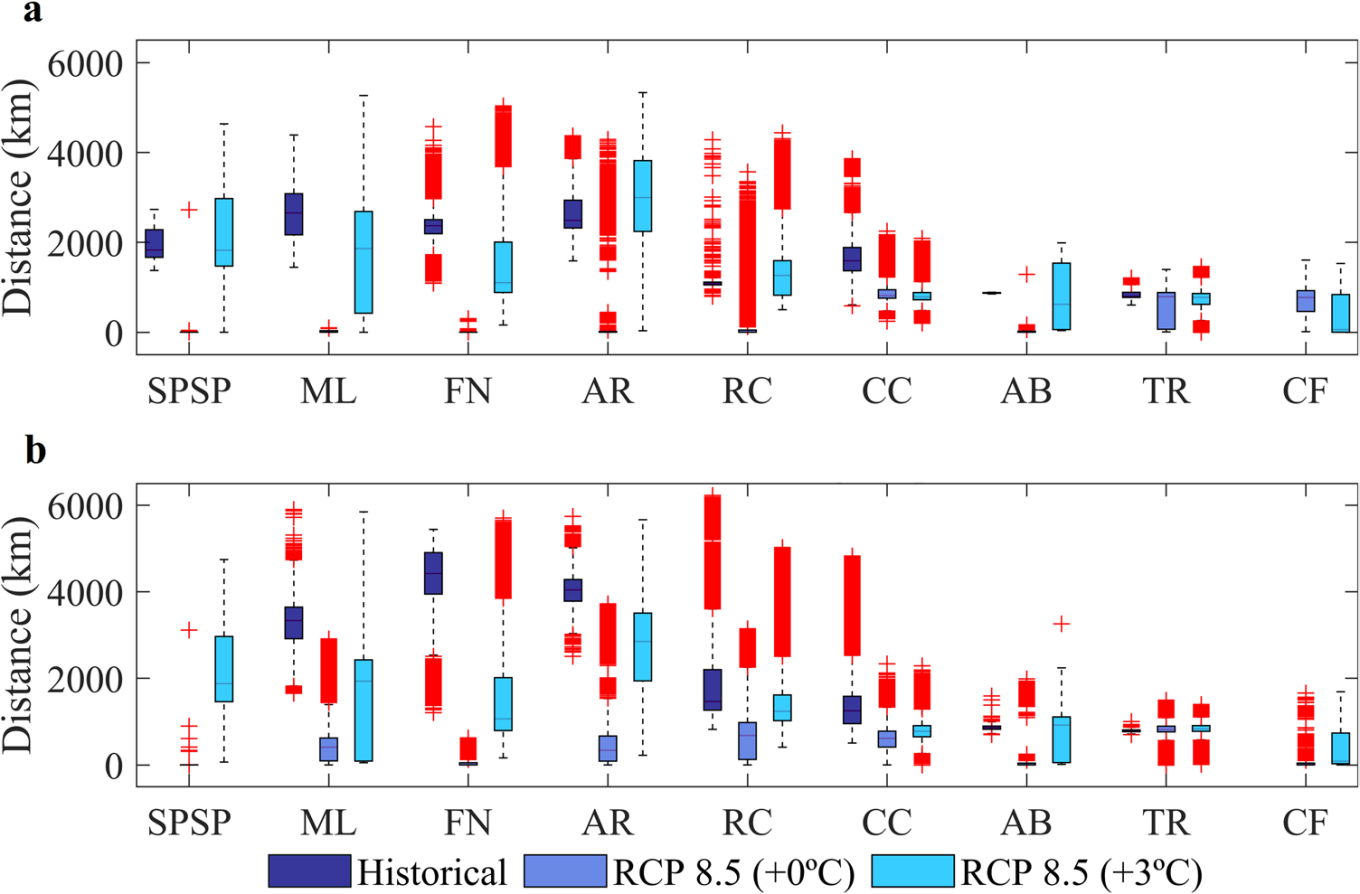
Boxplots of the average distances travelled by larvae for the summer (**a**) and winter (**b**) experiments for historical (2008–2015)^44^ (dark blue) and RCP 8.5 (2092–2099), considering non acclimated (+ 0 °C) (light blue), and acclimated (+ 3 °C) (turquoise) *Sparisoma* eggs and larvae. Red crosses represent the outliers of boxplots. Spawning (MPA) sites are: São Pedro and São Paulo Archipelago (SPSP), Parcel do Manuel Luis (ML), Fernando de Noronha Archipelago (FN), Atol das Rocas (AR), Recife dos Corais (RC), Costa dos Corais (CC), Abrolhos (AB), Trindade and Martim Vaz islands (TR), and Cabo Frio (CF). Created with Matlab R2019a (www.mathworks.com).

We compared the average distances travelled by larvae that were still alive at the end of historical^44^, non-acclimated and acclimated (this study) simulations (Fig. 2) to assess the influence of changes in ocean circulation on connectivities. Non-acclimated larvae released within the nSEC domain (sites 1,3 and 4 in Fig. 1 and SPSP, FN and AR, respectively, in Fig. 2a) are expected to travel shorter distances under RCP 8.5 than today but acclimated larvae will eventually travel distances comparable to the historical simulation during the summer (SPSP and AR in Fig. 2a). As the NBC retroflection becomes weaker in the future, both non-acclimated and acclimated larvae released in site 2 will travel shorter distances (ML in Fig. 2). We also show that between 5° S and 10° S the North Brazil Subcurrent (NBSC) will become weaker (− 0.4 m s^-1^) than today and the NBC will get stronger (around 0.2 m s^−1^) to the north of 5° S^45^. For sites 5 and 6 located in the NBSC, larvae will disperse for shorter distances in the future (RC and CC in Fig. 2) with the exception of acclimated larvae released in 6 during the summer (RC in Fig. 2a). The BC, flowing to the south, is also expected to become more intense (especially in the summer) southwards of 15° S (0.2–0.3 m s^−1^) (Fig. 1c). However, shelf flow will become slower (blue tones in Fig. 1c,d) and non-acclimated larvae released in site 7 is expected to travel shorter distances under RCP 8.5 than they experience today (AB in Fig. 2, note that no larvae survived in site 9 during historical simulations). The offshore island of TR (site 8 in Fig. 1) is located in a region of the southwest Atlantic where minimal changes is surface flow are expected for the RCP 8.5 scenario and the average distance travelled by larvae is likely to remain similar to today (TR in Fig. 2). The consequences of the heterogeneous spatial and seasonal warming and current patterns in the tropical Atlantic to total particle mortality and MPAs connectivity will be assessed in detail in the “Discussion” section.

### Egg and larvae mortality under extreme ocean warming

We simulated fish spawning, egg and larval dispersal and settlement in all MPAs considering the impacts of temperature and surface flow on mortality (lethal temperature and advection, respectivelly), for non-acclimated and acclimated eggs and larvae, assuming a maximum + 3 °C known thermal tolerance^46^ relative to present day. The lagrangian transport was computed using the model Ichthyop (version 3.3^47^) forced with hourly ocean conditions produced by the ocean (ROMS) downscaling of the RCP 8.5 scenario (Supplementary Fig. 3). Despite the coarse horizontal resolution of the native model (HadGEM2-ES, see “Methods”), the downscaling procedure resolved the ocean mesoscale dynamics to a resolution of approximately 9.3 km, consistent with the size of MPAs. The reef fish used in simulations is the grazer parrotfish of the genus *Sparisoma* that is widely distributed in reef areas along the Brazilian coast and oceanic islands^48^. The biological characteristics of *Sparisoma* necessary to run all simulations were obtained from the literature (Supplementary Table 2). A total of 70,000 eggs were released and advected for 60 days every summer (Jan–Feb) and winter (Jul–Aug), from 2092 to 2099, with quantities proportionally distributed among MPAs according to their size. We computed mortality due to lethal temperature (either high or low), and to particle advection outside the model domain, with their sum representing the total mortality (%). We have not considered the recent increase in MPA area determined for São Pedro e São Paulo Archipelago and Trindade Archipelago^49^, because it has not added new reef/hard substrate that could be used for recruitment.

**Figure 3:**
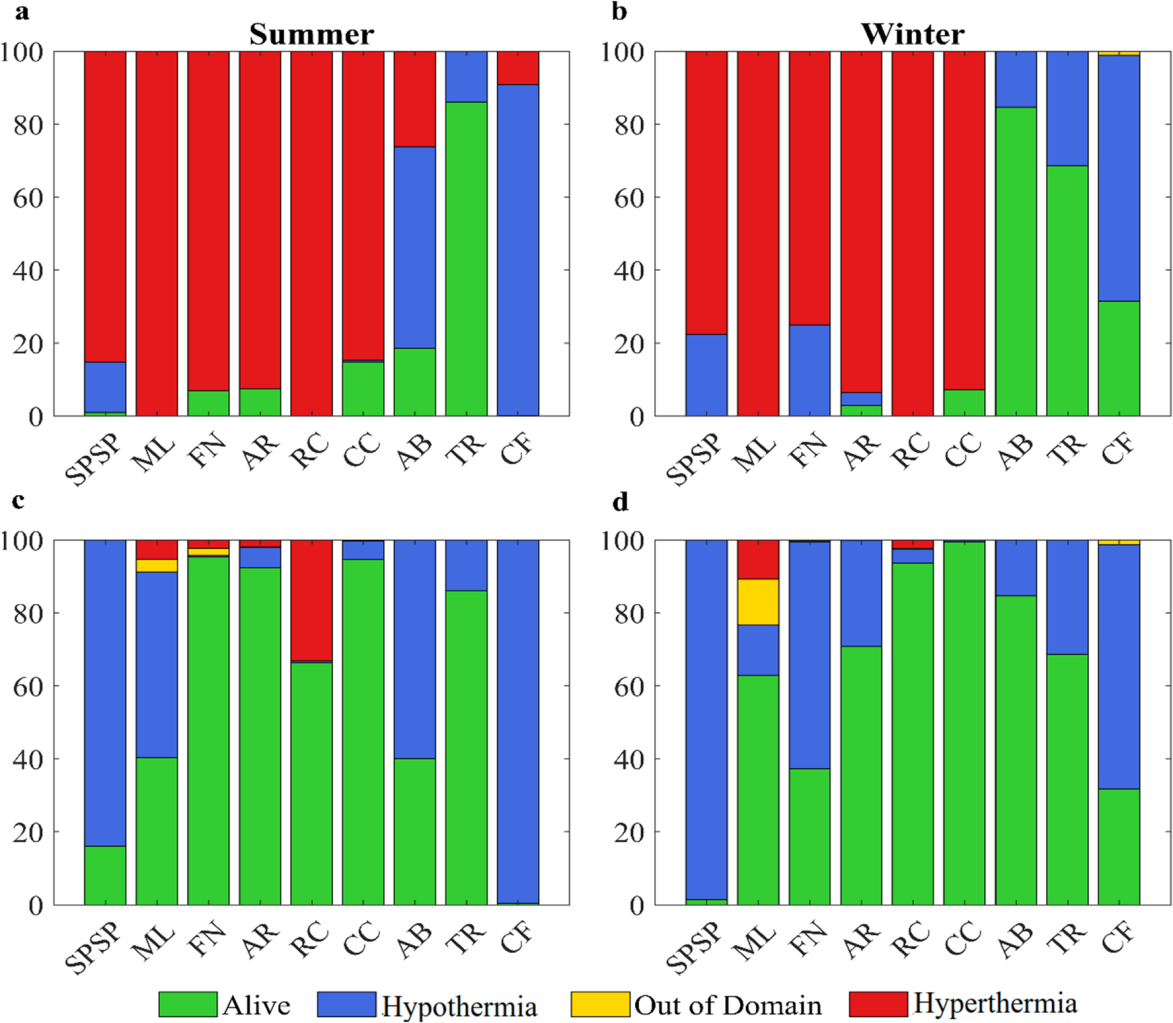
Proportion of particles spawned in each MPA that were alive or dead by hypothermia, hyperthermia or advected out of the domain at the end of simulations. (**a**) summer and (**b**) winter non-acclimated simulations (thermal tolerance between 24 and 30 °C); (**c**) summer and (**d**) winter acclimated simulations (thermal tolerance between 24 and 33 °C). Created with Matlab R2019a (www.mathworks.com).

Average temperatures for future climate change scenario in MPAs are 3 °C higher during the summer and 4 °C during the winter relative to the reference historical scenario (1997 to 2004). Lethal high temperature (hyperthermia) was the leading cause of mortality for non-acclimated particles in future scenarios (Fig. 3a,b), killing over 80% of the eggs in the first hours after spawning. In some MPAs, such as the northernmost ML and the more coastal RC, all eggs died after spawning both during the summer and winter. Mortality by lethal low temperature (hypothermia) also occurred in simulations. However, it was generally confined to the southernmost MPAs and in the equatorial SPSP, which is exposed to negative SST anomalies of the equatorial cold tongue. It should be noted that the lethal temperature range used here is comparable across *Sparisoma* species (see references used in Supp. Table 2). Mortality by advection of particles outside the model domain was close to zero but slightly higher in the acclimated simulations.

Increased tolerance to warmer ocean conditions in the acclimation simulations (Fig. 3c,d) led to lower mortalities and lowered interannual variability (not shown). The proximity to strong currents of the northernmost reefs of ML and high particle survival contributed to increasing the likelihood of a particle to leave the model domain. During the summer (winter), acclimation allowed the survival of 40.3% (62.9%) in ML and 66.3% (93.6%) in RC, contrasting with the complete die-off observed in the non-acclimated simulations. Acclimation in the southernmost protected coastal areas of AB and CF only marginally enhanced survival (around 2%). Total mortalities were significantly different between the acclimated and non-acclimated experiments (*p* < 0.001 by Kruskal–Wallis test), but differences between summer *vs* winter for the same thermal tolerance were not significant.

### Anticipated changes in demographic connectivity of MPAs

**Figure 4:**
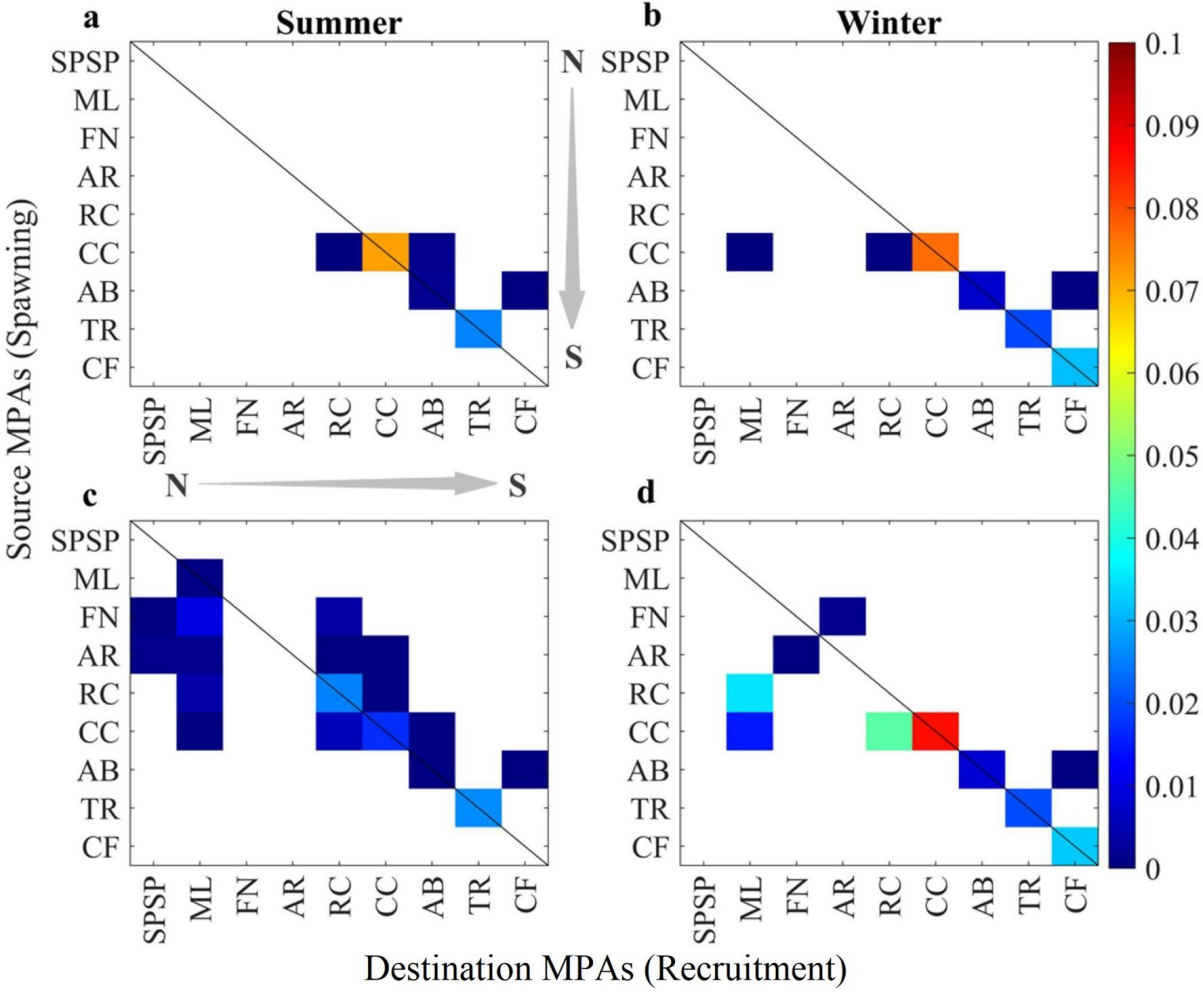
Seasonal Transition Probability Matrices (TPM) between source and recipient MPAs representing their demographic connectivity. Values along the diagonal black line indicate the probability of local retention of each MPA. Summer and winter connectivity are shown for non-acclimated (**a**,**b**) and acclimated (**c**,**d**) climate change simulations. Created with Matlab R2019a (www.mathworks.com).

The demographic connectivity of MPAs was computed as the probability that a particle leaving one site will be retained in the same site or reach a different one within its life span in the plankton (the pelagic larval duration, PLD). Larval recruitment and local retention rates were calculated for every summer and winter of each year and are summarised in the transition probability matrices (TPMs) shown in Fig. 4 (see “Methods” for details). Mortality caused by ocean warming has a strong influence on connectivity and will be higher without acclimation, especially in the egg phase. In some MPAs (ML, FN, AR, and RC), extreme die-offs will impede recruitment and their demographic connectivity. So, the closer an MPA is to the equator, the less connected it will be with the rest of the network (Fig. 4a,b).

The survival of eggs and larvae caused by acclimation will enhance the connectivity of MPAs in the northern half of the Southwestern Atlantic (Fig. 4c,d). Summer spawning will be especiallyimportant for the northernmost site of ML as it will receive larvae from FN and AR oceanic islands and the coastal reefs of RC and CC. An essential feature of the acclimation scenario is that the oceanic islands of FN and AR will become sources of larvae for coastal MPAs at the centre of the network such as RC and CC during the summer but not during the winter. It is also clear that CC will play a considerable role as a source of propagules for both southern and northern MPAs of RC and ML, respectively. Self-seeding induced by local retention (diagonal lines in Fig. 4) will be an important process for the maintenance of local populations of *Sparisoma* in the future for most coastal MPAs even without acclimation. Local retention is highest for CC and the southernmost CF in most scenarios, particularly during the winter.

## Discussion

Our study presents the potential impacts of global warming on the connectivity of a large network of MPAs extending along 4300 km in the Southwestern Atlantic. By not running simulations for mitigated emissions (e.g., RCP 4.5) we deliberately focus on the expected consequences of a worst-case (extreme) scenario. The changes reported here should, therefore, be viewed as a possible representation of the impact of some anthropogenic factors that influencing on the connectivity of MPAs. These impacts are anticipated by simulations on a biophysical modelling scheme integrating organismal (biological and behavioural) responses and the physical environment using a high-resolution ocean model. The main expected impact is a possible range expansion of *Sparisoma* dispersion towards subtropical reef environments facilitated by increasing directional agreement between ocean currents and temperature gradients^50, 51^. Seasonal changes in ocean temperature and surface currents generated by our ocean downscaling (Fig. 1) agrees with other CMIP5 projections of a stronger-than-present offshore BC alongside a weaker flow over the shelf south of 15°S and a weaker NBSC to the north^45^. Our downscaling experiments also revealed that seasonal warming will be largest during the winter (Fig. 1b), reducing mortality by hypothermia in CC, AB, TR and CF (also impacting total mortality, see below) seen in historical (2008–2015) simulations^44^. This differential seasonal warming usually goes undetected when SSTs are averaged over the region.

**Figure 5:**
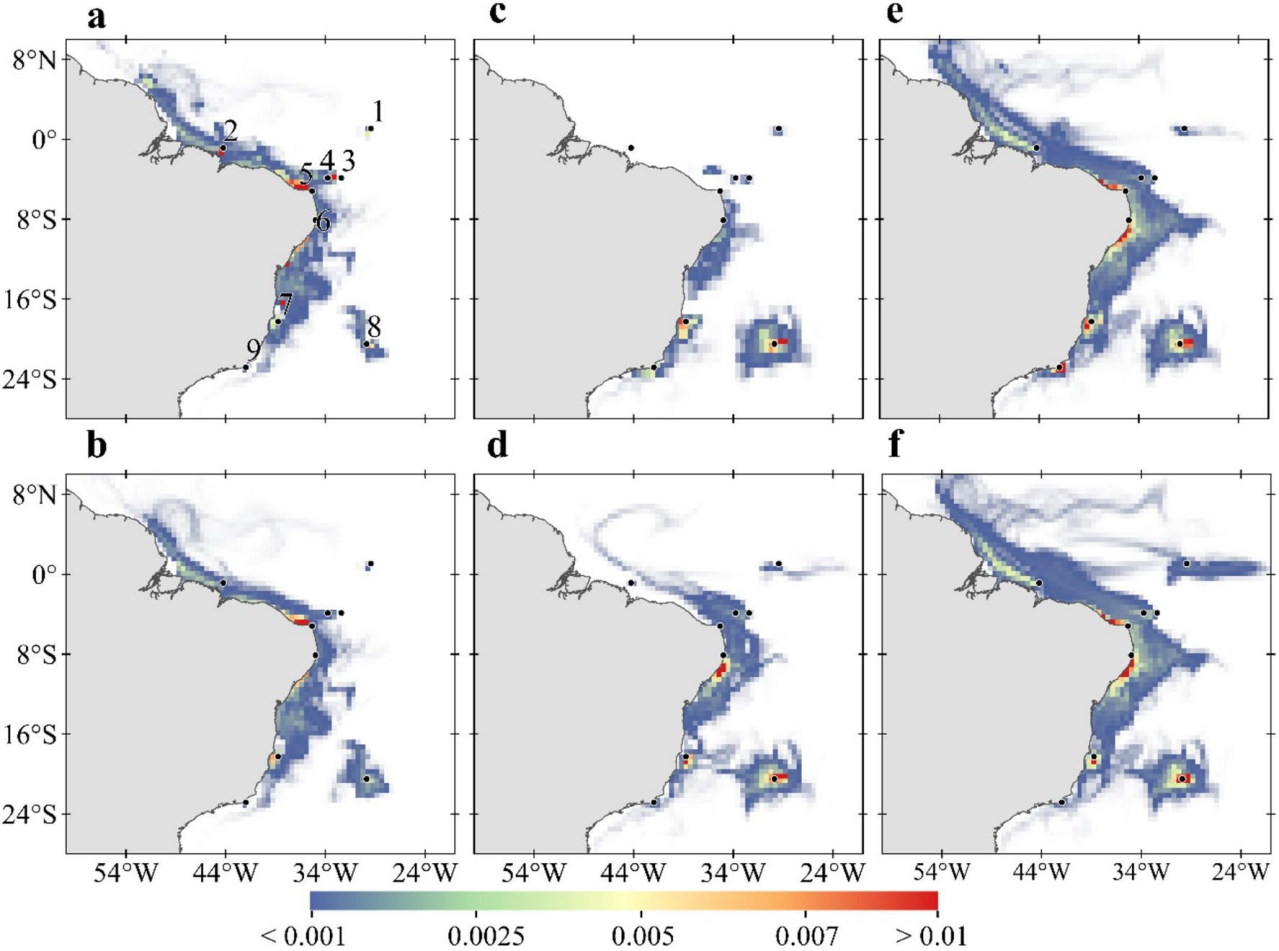
Spatial distribution of total surviving larvae during all experiments. (**a**) Summer and (**b**) winter based on present-day simulation (2008 to 2015). (**c**) Summer and (**d**) winter for RCP 8.5 (2092 to 2100) considering present-day thermal tolerance. (**e**) Summer and (**f**) winter considering acclimation of + 3 °C in RCP 8.5 scenario. Warm colours represent regions with the highest density of larvae along the simulations. Black dots represent MPA locations as in Fig. 1. Created with Matlab R2019a (www.mathworks.com) and QGIS-3.4 (https://www.qgis.org).

Different latitudinal mortality rates will enhance the isolation of island and coastal MPAs to the north (compare Fig. 5a,b and c,d), especially in the summer, caused by higher equatorward mortality resulting from ocean warming. Summer SSTs under climate change scenario will be warmer than today and above the tolerance threshold for *Sparisoma* in most MPAs, except for the southernmost sites (Supplementary Fig. 4). Results of biophysical simulations may vary depending on the numerical model used,but errors and biases of our ocean model (Supp. Fig. 5–8) are well constrained and within the range of variation of the biological model and do not compromise our interpretations. Besides, despite known SST biases in the tropical Atlantic, our results are in line with other climate models capturing the dominant seasonal variability^52^.

Ocean warming will cause higher total mortality of eggs and larvae of parrotfishes in northernmost MPAs (mostly in ML, FN, AR, RC and CC) compared with modelled present-day (historical simulation) results obtained from a different study using the same simulation approach^44^ (Supplementary Fig. 9). Note that for the southern MPAs of AB, CF and TR, simulated mortalities are higher today, in this case, caused by hypothermia, than our projection for the RCP 8.5 scenario. This future increase in larval survival will favour the tropicalization of the southernmost MPAs by a combination of mortality reduction of eggs and larvae contributing to local retention and a north–south connection of CC-AB (summer only) and AB-CF (summer and winter, Fig. 4a,b).

We can infer from our results how larval mortality, reproductive timing, and changes in hydrodynamics will impact the effectiveness of MPA networks in the future^53, 54^. Note that the biological impact of future changes is limited to fish survival (i.e., mortality) and fisiological changes were not directly simulated, although we did include the possibility of acclimation. Without acclimation, total mortality for summer spawning will be higher than today for most MPAs, but much lower during the winter for the southern AB and CF (Supp. Figure 9b), probably impairing summertime spawning and limiting the reproductive season to winter. Six out of nine MPAs are in the warmest tropical sites, where local loss is unlikely to be replaced^55^. We show that connectivity under a warmer tropical Atlantic will not only shift southwards, but it will also increase local retention at these southern MPAs (Fig. 4a,b). This finding contrasts with the low present-day demographic connectivity and local retention in these MPAs determined for *Sparisoma*^44^. Annual recruitment rates under RCP 8.5 conditions (Supplementary Fig. 10) will be reduced by one order of magnitude compared to present-day simulations. However, the number of MPAs with local retention (calculated in the same way as self-recruitment in^44^) will increase (Supplementary Fig. 11) from a maximum of two sites today (CC and TR) to four (CC, AB, TR and CF) in the future. This increase in local retention is the result of an increase in larval survival in AB and CF due to warmer conditions compared to simulations carried out for present conditions (see Fig. 2 in^44^) and weaker coastal flow (Fig. 1c,d).

During the summer, acclimation will potentially increase the island-to-shelf demographic connectivity, between the oceanic islands of FN, AR and ML in the northern shelf. A southerly oriented pathway will also connect the islands FN and AR with the coastal RC and CC, forming another important island/shelf network (Fig. 4c). This connectivity of acclimated propagules spawned at FN and AR with ML may be related to an increase in survival (Supplementary Fig. 9) and the likelyhood of entering the stronger nSEC under RCP 8.5 scenario. During the winter, the FN-AR connection with the coastal RC and CC sites will be lost due to higher mortalities (Supp. Figure 9b) and the seasonal southward migration of the sSEC bifurcation. Our simulations show a general pattern characterized by a consistent southward shift of connectivity under severe ocean warming relative to today^44^. Without acclimation, the potential spatial distribution of particles in the future will be shifted southwards during the summer, because of excessive mortality at lower latitudes (e.g., ML and RC). This is highlighted by the spatial distribution of surviving larvae that shows a tendency for isolation of oceanic islands and coastal MPAs during the summer, with northernmost (1, 2, 3 and 4 in Fig. 5c) and southernmost MPAs (7, 8 and 9 in Fig. 5c) disconnected from the central region. Connectivity will also shift towards the south compared to the present-day. However, the spatial distribution of non-acclimated larvae during the winter is not as fragmented (see Fig. 5d). Without acclimation, there is a risk that connectivity of tropical MPAs in the south Atlantic could be further reduced or even lost under a climate change scenario. For coastal areas, larval dispersion patterns suggest that increasing the extent of protected recruitment areas or adapting MPAs boundaries could minimize this loss.

We have shown that ocean warming may cause detectable changes in MPA connectivity, including the reduction in the incidence of fish larvae at northernmost MPAs, isolation of oceanic islands and a connectivity shift of the network towards the south (Fig. 5a to d). On the other hand, acclimation will not only allow high particle survival but also enhance their spatial distribution and increase their incidence in tropical and southern MPAs (Fig. 5e,f). Another critical difference between present-day connectivity and those simulated for a warmer ocean is an increase of local retention for both non-acclimated and acclimated reef fish propagules. Both low mortality and weaker shelf currents play a role in the increase of local retention in coastal MPAs such as CC, AB and CF for the RCP 8.5 simulations. Note that in these sites eggs and larvae will tend to travel shorter distances compared to those in more energetic sites like FN and AR (See Fig. 2). One of the most fragile MPA is TR because, as today, local retention is the only source of recruits (site8 in Fig. 5). The high concentration of larvae around TR seen in Fig. 5 suggests that local retention may be influenced by the physical interaction of the flow with the Vitória-Trindade seamount chain reducing local advection. These features are adequately represented in the eddy-resolving (high horizontal resolution) model used in the dynamical downscaling of the RCP 8.5 scenario.

**Figure 6:**
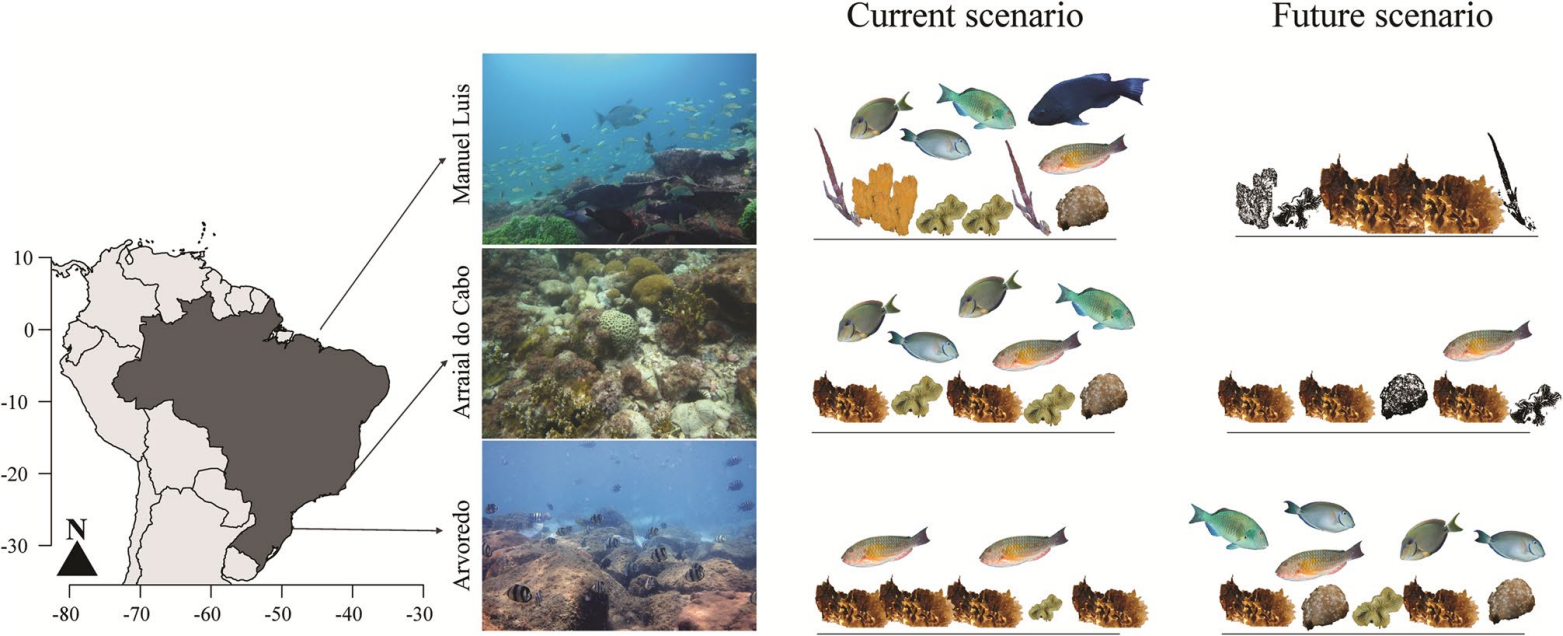
Climate-change induced tropicalization of subtropical and temperate reefs. Schematic representation of the projected changes in reef ecosystems of the western south Atlantic (first column) caused by tropicalization. The second and third columns depict the present-day higher biomass of herbivore reef fish in the tropics^56^ and higher macroalgae and turf algae biomass in the subtropic and temperate sites^57^. The future climate change scenario is depicted in the fourth column with an increase of algal cover in tropical reefs as a response to greater mortality of grazers and reduced grazing pressure. Subtropical to temperate reefs will display higher grazer fish biomass and lower dominance of macroalgae. Created with Adobe Illustrator-2020 (https://www.adobe.com/).

Tropical MPAs around the world are likely to become increasingly exposed to extreme thermal conditions and future conservation decisions will demand the understanding of complex biophysical interactions. The interplay of temperature-induced reef fish mortality and regional ocean circulation suggests that connectivity may become seriously compromised in low latitude habitats but enhanced in temperate ones. The poleward movement of reef fauna under global warming driven by the search for suitable thermal environments may occur concomitantly with acclimation due to transgeneracional metabolic acclimation^13, 33^. The fact that the same reef fish species can be found today living at different latitudes and temperature regimes (e.g., *S. radians*, 33°N–30°S) suggests that acclimation over two or more generations may become an important adaptation to ocean warming in the future. The tropicalization of subtropical and temperate MPAs raises significant concerns about their functional integrity and long-term resilience since the arrival of warm species has the potential to alter ecological network structure and lead to a complete ecosystem transformation (Fig. 6). In this regard, the major losses of marine diversity and biomass of herbivores fishes in the tropics and gains in subtropical areas suggest that these will experience substantial species turnover and homogenization.

## Methods

### Hydrodynamical model

The regional ocean downscaling seeks to combine large-scale atmospheric features and global climate variability from Earth System Models (ESMs) with smaller-scale local conditions, especially mesoscale ocean activity, resolved in high-resolution regional models. Along this process temporal, horizontal and vertical resolutions from ESMs are numerically interpolated to a higher resolution grid. We used both historical simulation and RCP 8.5 projections from the Earth System Model HadGEM2-ES from CMIP5, ensemble r2i1p1 (available at https://esgf-data.dkrz.de/projects/esgf-dkrz/^39^) as our source of global climate variability. This ensemble was chosen because it provides all the atmospheric and ocean variables required by the ocean model (see Supplementary Table 1) to run the downscaled experiments (initial, forcing and boundary conditions) and has been used in the region with good results^58, 59^. The historical simulations were used to assess the likely root mean square error and bias of the RCP 8.5 ocean downscaling experiment. The atmospheric resolution of the forcing variables is 1.875° × 1.25° with 38 vertical levels and an ocean resolution of 1° (increasing to 1/3° at the equator), with 40 vertical levels^39^. The forcing and ocean boundary conditions were applied to the Regional Ocean Modelling System (ROMS, version 3.7) developed by Rutgers^36^ for the dynamical downscaling.

The ocean grid domain of the downscaling simulations is bounded at 10° N–30° S and 20° W–70° W with a horizontal resolution of 1/12° (approximately 9.3 km) and 30 vertical sigma levels. Hourly outputs of the downscaling experiment at each grid point and depth layer were stored every year for January–February (summer) and July–August (winter), to be used in the biological simulations. Air-sea fluxes were calculated using the bulk parameterization^60, 61^, and the open boundary conditions were defined according to the previous work^62^. A Flather-type condition was applied for the barotropic mode, the free surface, and the mixing of turbulent kinetic energy (TKE). The Chapman-implicit boundary condition was applied to the closed boundaries. The baroclinic mode and the inertial passive tracers used a combination of radiation and nudging^63^ and the vertical mixing parameterization was the K-profile turbulence closure^64^. The model atmosphere was forced with HadGEM2-ES outputs, every six hours by surface forcing parameters and monthly by ocean boundary components. The list of these parameters is listed in Supplementary Table 1.

Two main simulations were performed, one called historical that was run from 1997 to 2004 and another called RCP 8.5 that spans the period between 2090 and 2100 (Supplementary Table 3). Results from historical simulation were compared with SST from Operational Sea Surface Temperature and Sea Ice Analysis (OSTIA, available at http://ghrsst-pp.metoffice.com/data/OSTIA^40^) and vertical temperature profiles from Prediction and Research Moored Array in the Tropical Atlantic buoys (PIRATA) (available at https://www.pmel.noaa.gov/tao/drupal/disdel/, Supplementary Fig. 12)^65^, surface currents from the Simple Ocean Data Assimilation (SODA, version 3.4.2, available at https://www.atmos.umd.edu/~ocean/index_files/soda3.4.2_mn_download_b.htm)^41^. Differences between RCP 8.5 and historical simulations were calculated to characterize the main hydrodynamical changes between them (Fig. 1). The dynamical downscaling solutions were compared with observations by computing the Root Mean Square Error (RMSE) (Supplementary Figs. 6, 8) and bias (Supplementary Figs. 5, 7):

1 $$RMSE=\sqrt{\frac{1}{N}{\sum }_{i=1}^{n}{\left({X}_{ROMS}-{X}_{obs}\right)}^{2}}$$

2 $$Bias=\frac{1}{N}{\sum }_{i=1}^{n}\left({X}_{ROMS}-{X}_{obs}\right)$$

Hourly outputs of austral summer (January–February) and winter (July–August) from RCP 8.5 (2092–2100) were stored to be used as an input for the biological simulations (Supplementary Fig. 3).

### Egg and larval transport

Egg and larval dispersion and settlement into MPAs of the reef fish genus *Sparisoma* (Scaridae) were simulated for a non-acclimated scenario assuming present-day thermal tolerance (24 to 30 °C) and an acclimated scenario where eggs and larvae are able to adapt to an increase of up to + 3 °C in SST. Simulations were performed using the Lagrangian advection model ICHTHYOP (version 3.3)^47^ based on hourly fields forcing conditions produced by the RCP 8.5 (2092–2100). This genus is found in the majority of reef areas along the Brazilian coast and islands^20^, although not always as a stable population. A total of 70,000 eggs were released and advected for 60 days in each experiment. The quantity launched for each MPA was proportionally distributed according to their size (Table 1). The recent increase in the MPA area determined for São Pedro e São Paulo Archipelago and Trindade^49^ was not considered as it did not result in an increase of reef substrate necessary for fish spawning and recruitment^66^.

Spawning can happen at any time during the year but tends to concentrate during summer and winter, and eggs are released in the first meters of the water column^67^. Therefore, in our simulations, spawning occurred yearly, both in summer (January and February) and winter (July and August) from 1997 to 2004 and from 2092 to 2100. The egg phase duration was set to 24 h, and egg density is 0.0089 g cm^−3^, following^68^. Each simulation had a duration of 60 days, equivalent to the average PLD found for this genus^69^. Particles were propagated over time using Runge–Kutta 4th Order scheme^70^ with a time step of 1 h.

Recruitment was computed as the total number of larvae inside an MPA with larvae considered competent to recruit only at the end of each simulation (Eq. 3). Local Retention (LR) represents the number of particles spawned in an MPA that remained in the same MPA (Eq. 4)^44^.

3 $$R_{t,i} = \frac{{\sum {}_{t}^{{}} c_{ij} + \sum {}_{t}^{{}} c_{ii} }}{{\sum {}_{t}^{{}} N_{s} }}$$

4 $$LR_{t,i} = \frac{{\sum {}_{t}^{{}} cii}}{{\sum {}_{t}^{{}} N_{i} }}$$

where* t* is the integration of time for each simulation; c_*ij*_ represents the larvae spawned in MPA *i* that were recruited in MPA *j*; N_*s*_ is the total number of eggs spawned in all MPAs; c_*ii*_ is the number of particles spawned in MPA i that were recruited in the same MPA; *N*_*i*_ is the number of eggs spawned in MPA *i*.

The transition probability matrices (TPM), or simply connectivity matrices, were calculated to indicate the probability of a particle spawned in site *i* (source) to recruit in *j* (sink) or remain in *i*, at time *t*^53, 71^. A Kruskal–Wallis test was used to identify significant variabilty in mortalities among seasons and between the acclimated and non-acclimated simulations. The likelihood of larvae to cross each grid cell of the domain shown in Fig. 4 was estimated using the kernel density estimation method^72^, assembling all years of each simulated scenario. Lower survivorship tends to inflate the probability density so, to make these maps comparable, the final probabilities were scaled by giving more weight to simulations with larger numbers of living particles.

## Supplementary information

Supplementary Information.

## Data Availability

All data needed to evaluate the conclusions in the paper are present in the paper and the Supplementary Materials. Additional data related to this paper may be requested from the authors.
