# Supplementary material for: Potential changes in the connectivity of marine protected areas driven by extreme ocean warming
Source: Sci Rep. 2021 May 14;11:10339. doi: 10.1038/s41598-021-89192-6 (PMC8121921; doi:10.1038/s41598-021-89192-6)
Supplement: Supplementary file 1 — Supplementary Information. [file 41598_2021_89192_MOESM1_ESM.pdf]

Potential changes in the connectivity of marine protected areas driven by extreme ocean warming

Authors: Luciana S. Lima*, Douglas F.M. Gherardi, Luciano P. Pezzi, Leilane G. Passos, Clarissa A.K. Endo, Juan P. Quimbayo
*corresponding author (luciana.lima@inpe.br)

Supplementary Information

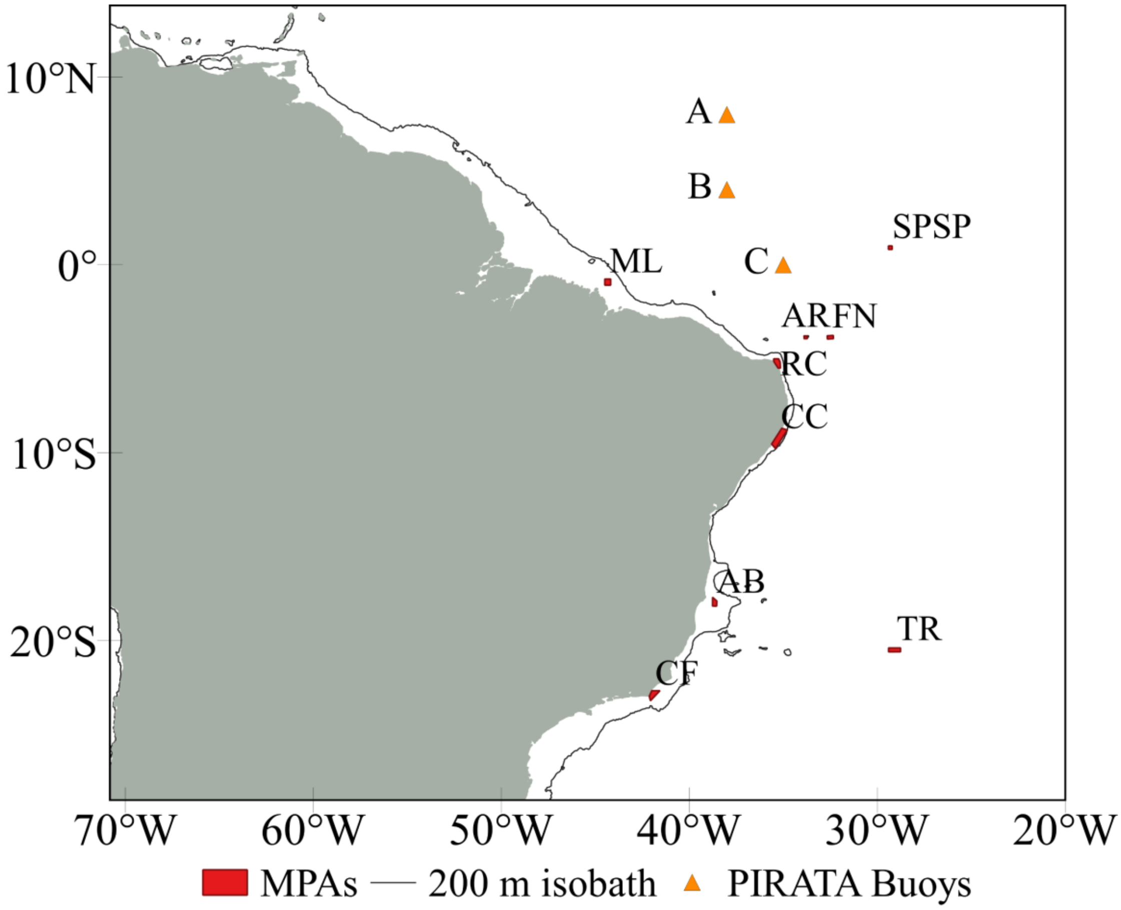

Supplementary Figure 1: Location of MPAs used as spawning and recruitment sites in the connectivity simulations. There are five MPAs on the continental shelf: Manuel Luís (ML), Recife de Corais (RC), Costa dos Corais (CC), Abrolhos (AB) and Arraial do Cabo and Cabo Frio region (CF), and four MPAs in oceanic island: Atol das Rocas (AR), Fernando de Noronha Archipelago (FN), Archipelago de São Pedro e São Paulo (SPSP) and Martim Vaz e Trindade Archipelago (TR). Prediction and Research Moored Array in the Tropical Atlantic (PIRATA) buoys location are: A) 0º N 35º W; B) 4º N 38º W; C) 8º N 38 ºW. Created with QGIS-3.4 (https://www.qgis.org).

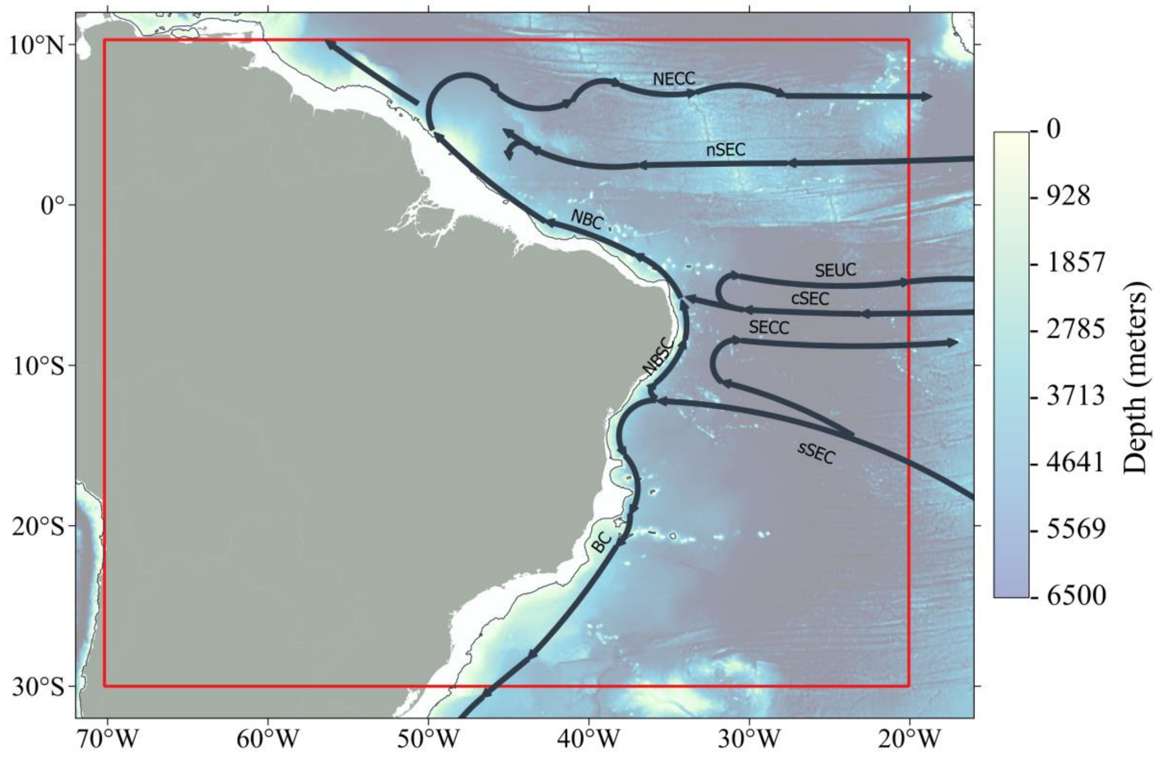

Supplementary Figure 2: Map showing the mean ocean surface flow and main currents in the tropical southwest Atlantic[1–3]. Brazil Current (BC); southern branch of the South Equatorial Current (sSEC); North Brazil Sub-Current (NBSC); South Equatorial Countercurrent (SECC); central branch of the South Equatorial Current (cSEC); South Equatorial Undercurrent (SEUC); North Brazil Current (NBC); northern branch of the South Equatorial Current (nSEC); North Equatorial Countercurrent (NECC). Red rectangle delimits the model domain. Created with QGIS-3.4 (https://www.qgis.org).

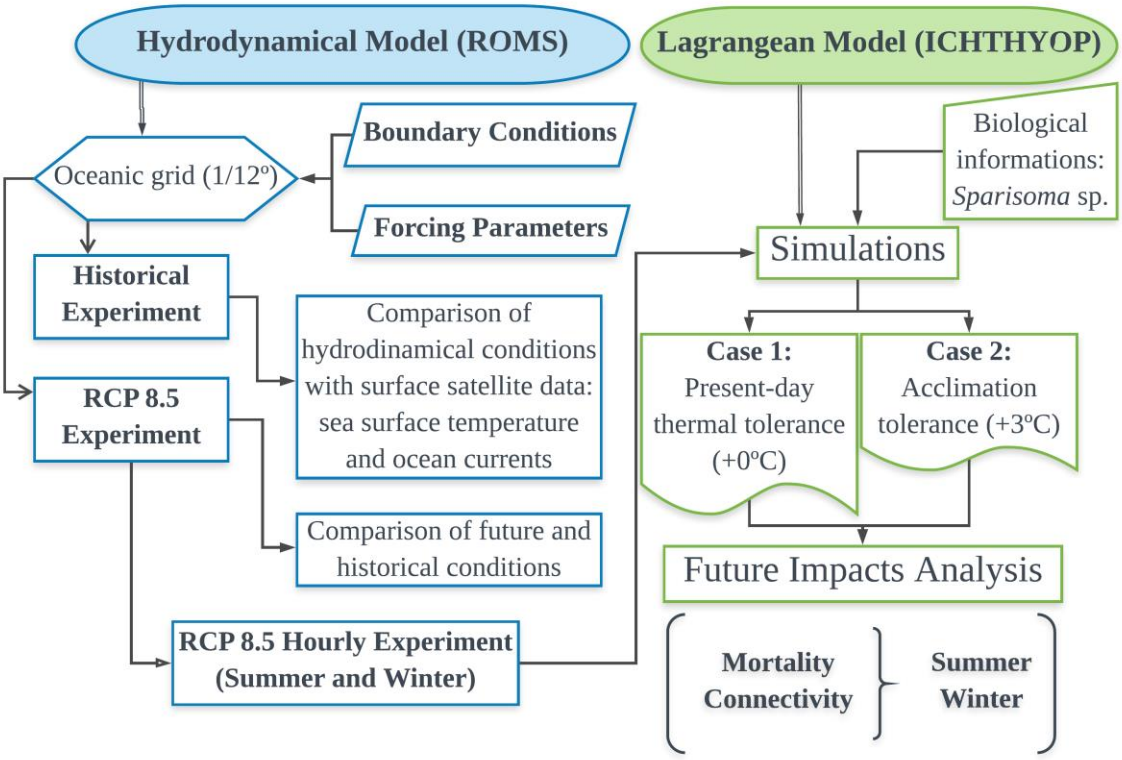

Supplementary Figure 3: Schematic Representation of hydrodynamical and biological simulations. Blue chart represents the hydrodynamical simulation steps: firstly, the boundary conditions and forcing parameters were prepared for the 1/12º grid downscaling historical experiment. Results of this simulation were compared with surface satellite data to verify the oceanic components. The same configuration was used to run the climate change RCP 8.5 experiment. Monthly dynamic downscaled RCP 8.5 oceanic fields were compared with historical results by computing bias and errors. The third oceanic simulation, the RCP 8.5 hourly experiments for summer and winter, was used as input to the Lagrangean biological simulations (green chart). We carried out two biological simulations: case 1: considering the actual thermal tolerance (24 ºC to 30 ºC); and case 2: allowing for the acclimation of +3 ºC above the present-day thermal tolerance (33 ºC) for eggs and larvae. The mortality and demographic connectivity among MPAs were computed for both summer and winter simulations.

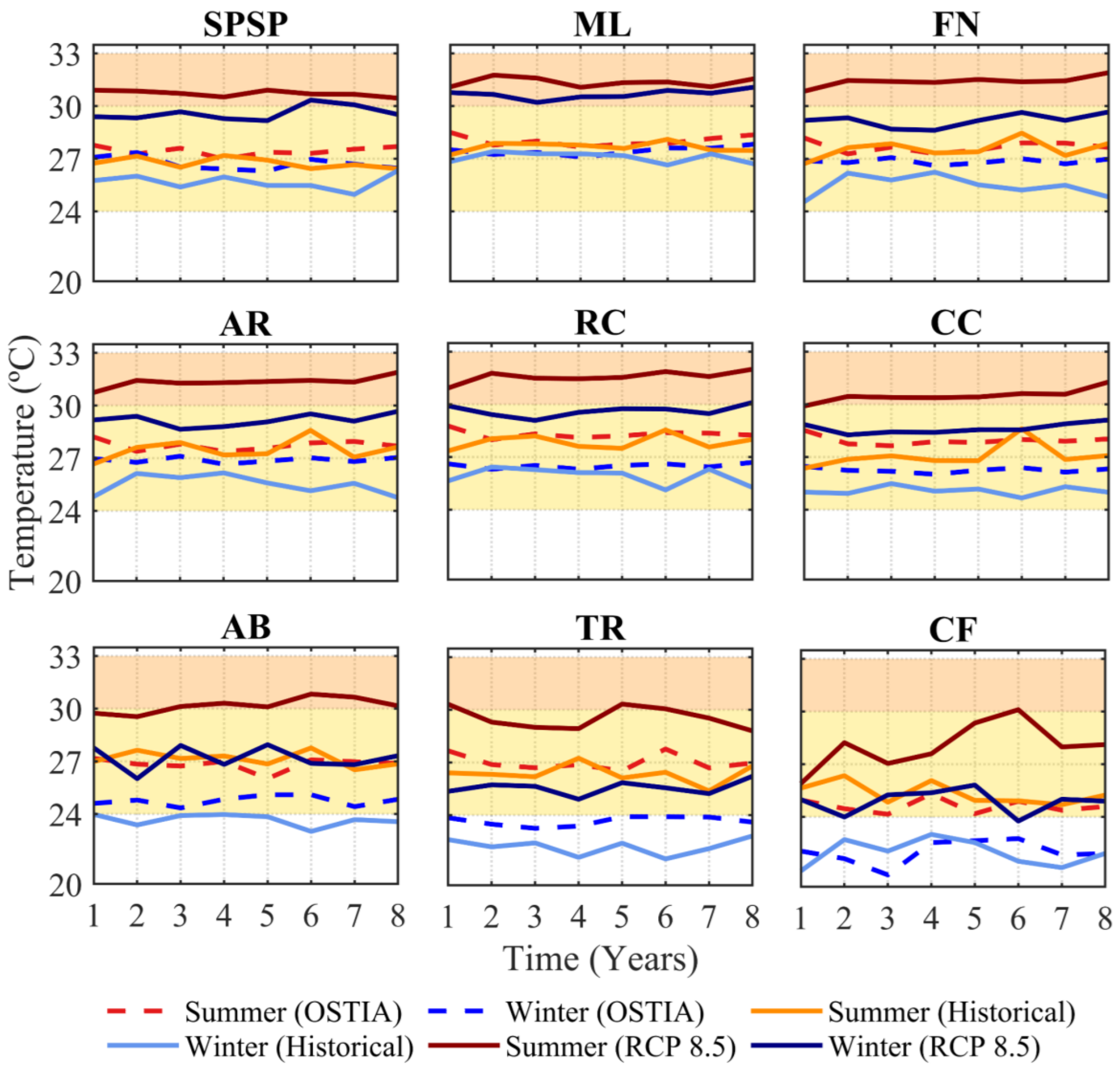

Supplementary Figure 4: Present and future mean sea surface temperatures in the marine protected areas. Present-day values (1997-2005) are from OSTIA (observation) and historical dynamical downscaling of HadGEM2-ES, future values (2092-2100) are from the dynamical downscaling of HadGEM2-ES RCP 8.5 calculated for austral summer (Jan-Feb-Mar) and winter (Jul-Aug-Sept). Present-day thermal tolerance (+0ºC) range for *Sparisoma* is indicated by the yellow stripe and the +3 °C acclimation range is indicated by the pale orange stripe. MPA abbreviations are the same as in Supplementary Figure 1. Created with Matlab R2019a (www.mathworks.com).

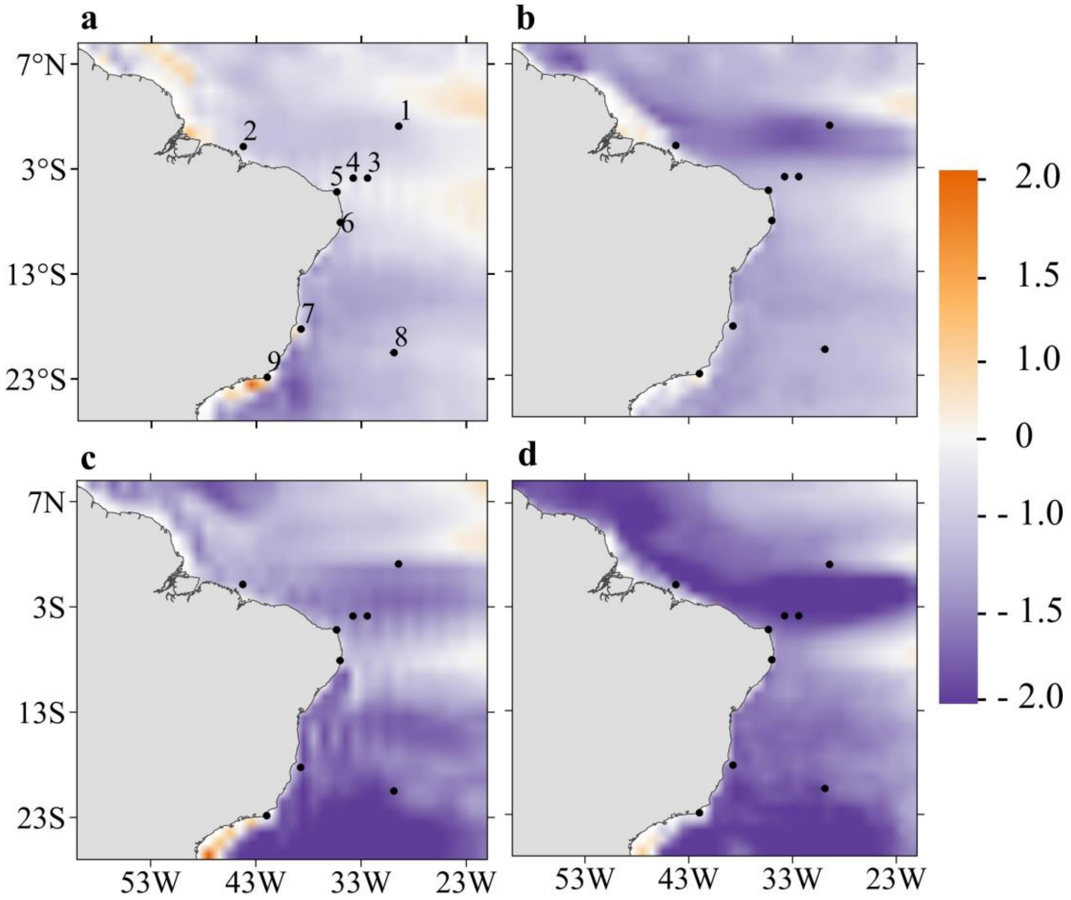

Supplementary Figure 5: Model mean sea surface temperature bias (ºC) relative to OSTIA. (a) HadGEM2-ES r2i1p1 historical austral summer (1997-2004); (b) ROMS (1997-2004) austral summer; (c) HadGEM2-ES r2i1p1 historical austral winter (1997-2004); (d) ROMS (1997-2004) austral winter. Created with Matlab R2019a (www.mathworks.com) and QGIS-3.4 (https://www.qgis.org).

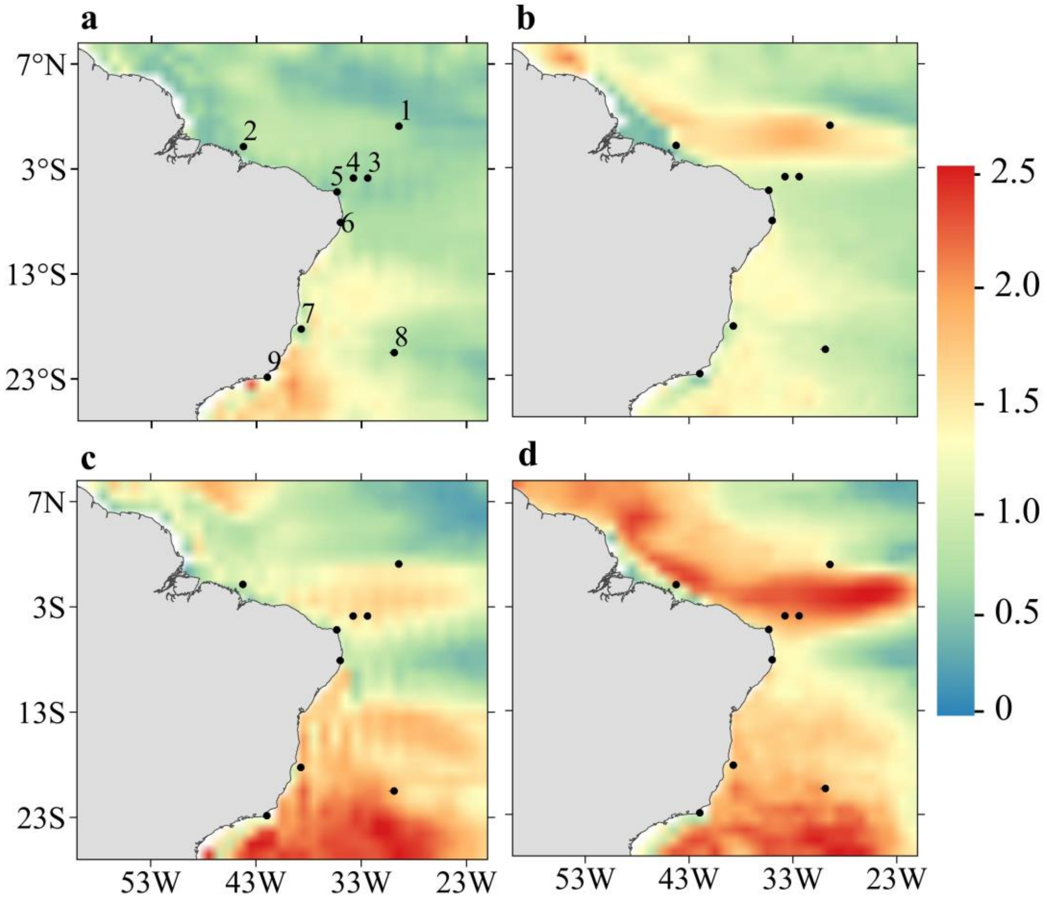

Supplementary Figure 6: Model mean sea surface temperature RMSD (ºC) relative to OSTIA. (a) HadGEM2-ES r2i1p1 historical austral summer (1997-2004); (b) ROMS (1997-2004) austral summer; (c) HadGEM2-ES r2i1p1 historical austral winter (1997-2004); (d) ROMS (1997-2004) austral winter. Created with Matlab R2019a (www.mathworks.com) and QGIS-3.4 (https://www.qgis.org).

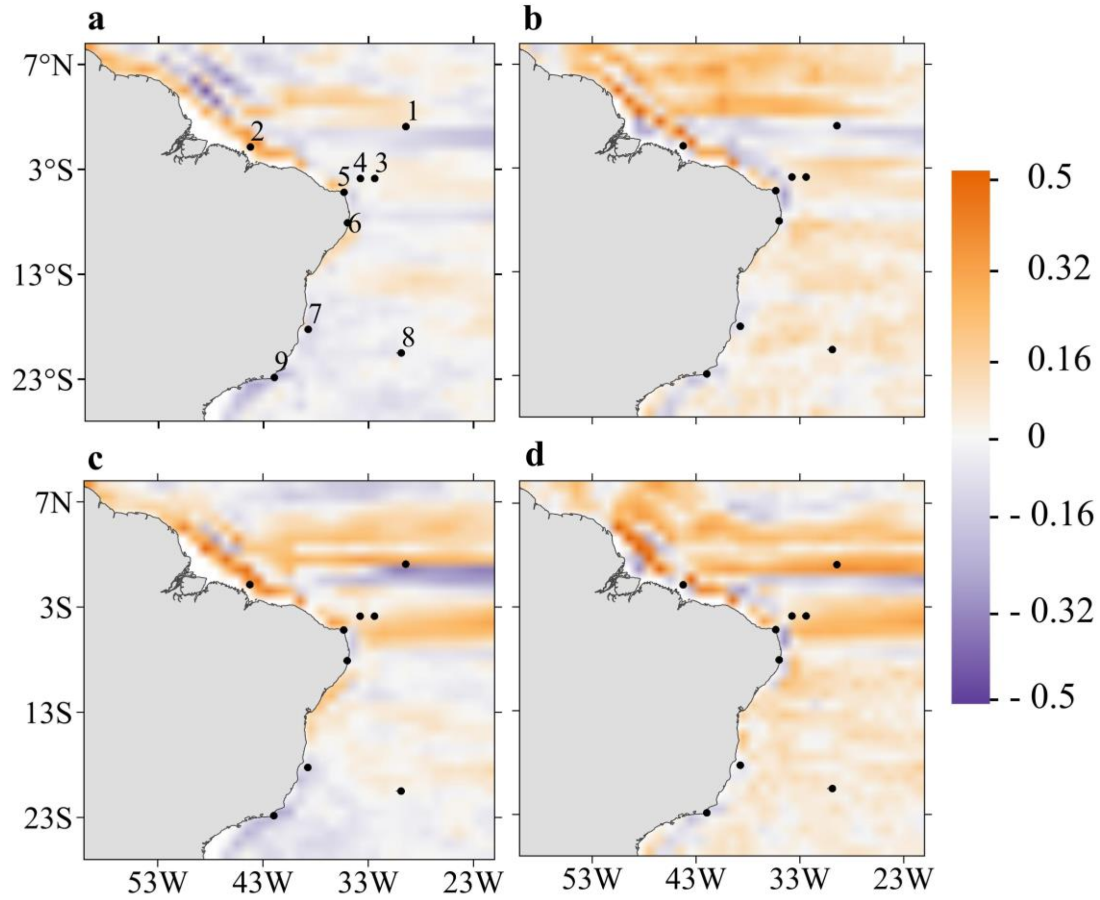

Supplementary Figure 7: Model mean surface currents (0-100m depth) bias (m s^{-1}) relative to SODA. (a) HadGEM2-ES r2i1p1 historical austral summer (1997-2004); (b) ROMS (1997-2004) austral summer; (c) HadGEM2-ES r2i1p1 historical austral winter (1997-2004); (d) ROMS (1997-2004) austral winter. Created with Matlab R2019a (www.mathworks.com) and QGIS-3.4 (https://www.qgis.org).

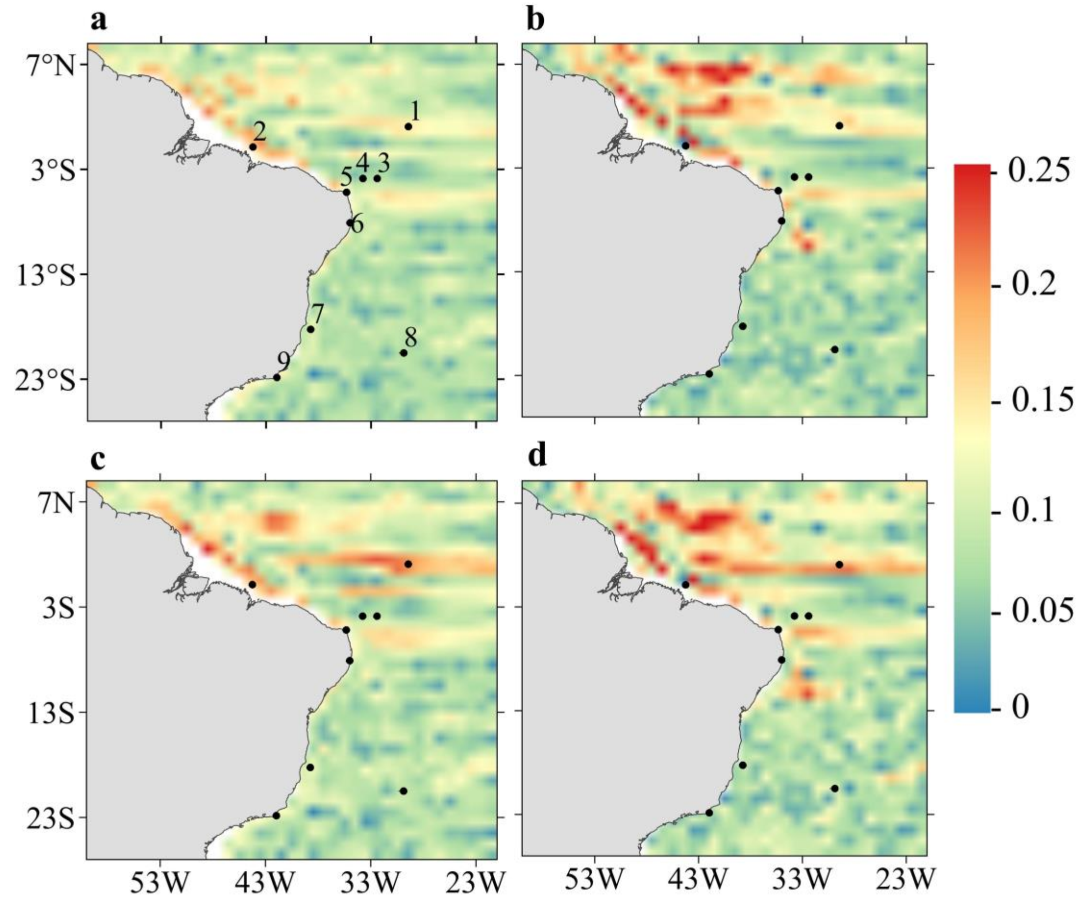

Supplementary Figure 8: Model mean surface currents (0-100m depth) RMSD (m s^{-1}), relative to SODA data. (a) HadGEM2-ES r2i1p1 historical austral summer (1997-2004); (b) ROMS (1997-2004) austral summer; (c) HadGEM2-ES r2i1p1 historical austral winter (1997-2004); (d) ROMS (1997-2004) austral winter. Created with Matlab R2019a (www.mathworks.com) and QGIS-3.4 (https://www.qgis.org).

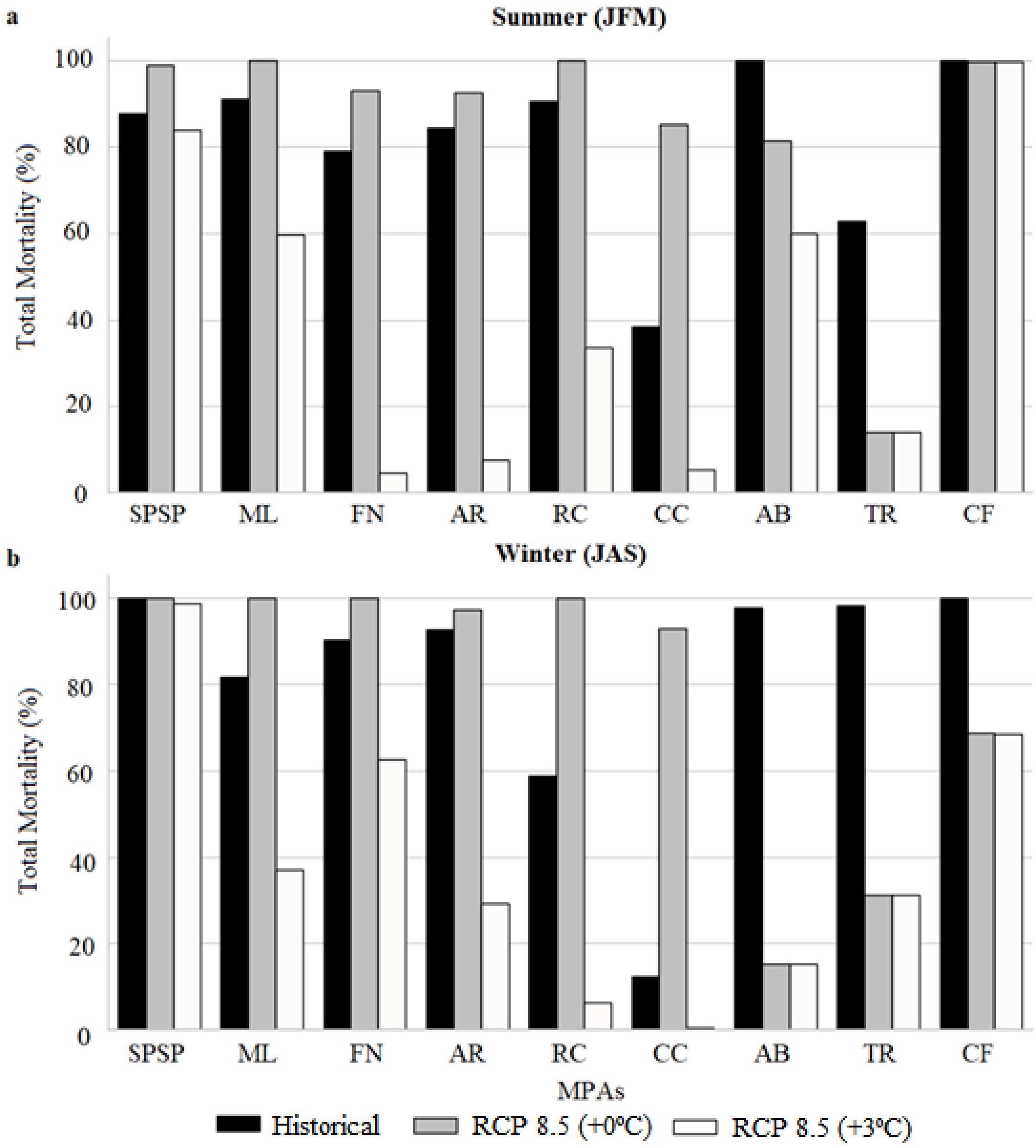

Supplementary Figure 9: Eight-years mean mortality per MPA for present-day and future conditions (+0ºC, non-acclimated and +3ºC, acclimated). Present-day mortality was calculated for 2008 – 2015[4], note that results are comparable because the ocean model used was the same (ROMS) and biological simulations were run with the same configuration, except for maximum lethal temperature. Gray and white bars represent the results of mean mortality considering the present-day thermal tolerance (gray) and acclimation of +3 ºC (white). (a) Summer; (b) Winter. Created with Python 2.7 using Seaborn library.

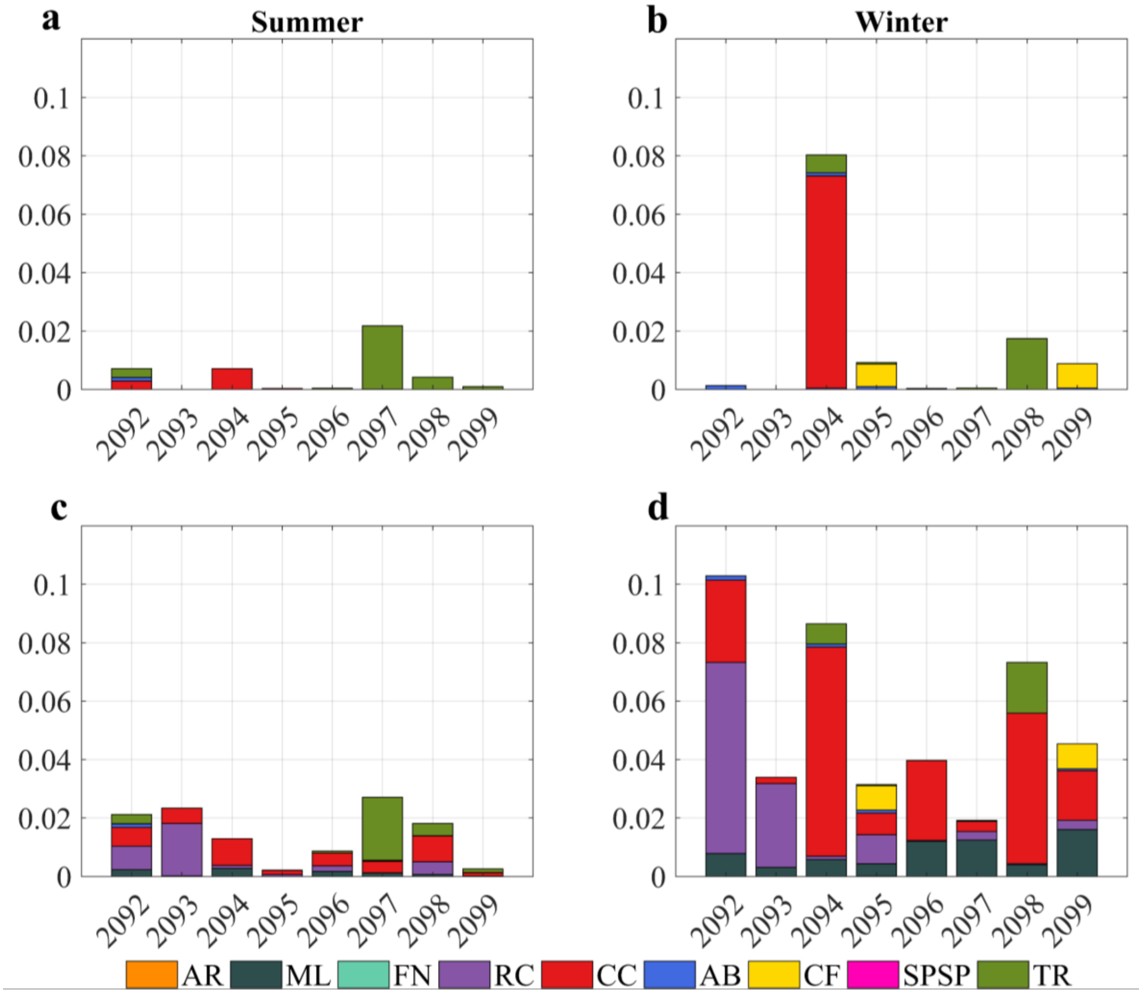

Supplementary Figure 10: Recruitment per year as a proportion of total spawned particles in each MPA considering the present-day thermal tolerance, in (a) summer and (b) winter; and considering acclimation of +3ºC in (c) summer and (d) winter. Each color represents the contribution per MPA in the total recruitment (recruitment and local retention) per year. Created with Matlab R2019a (www.mathworks.com).

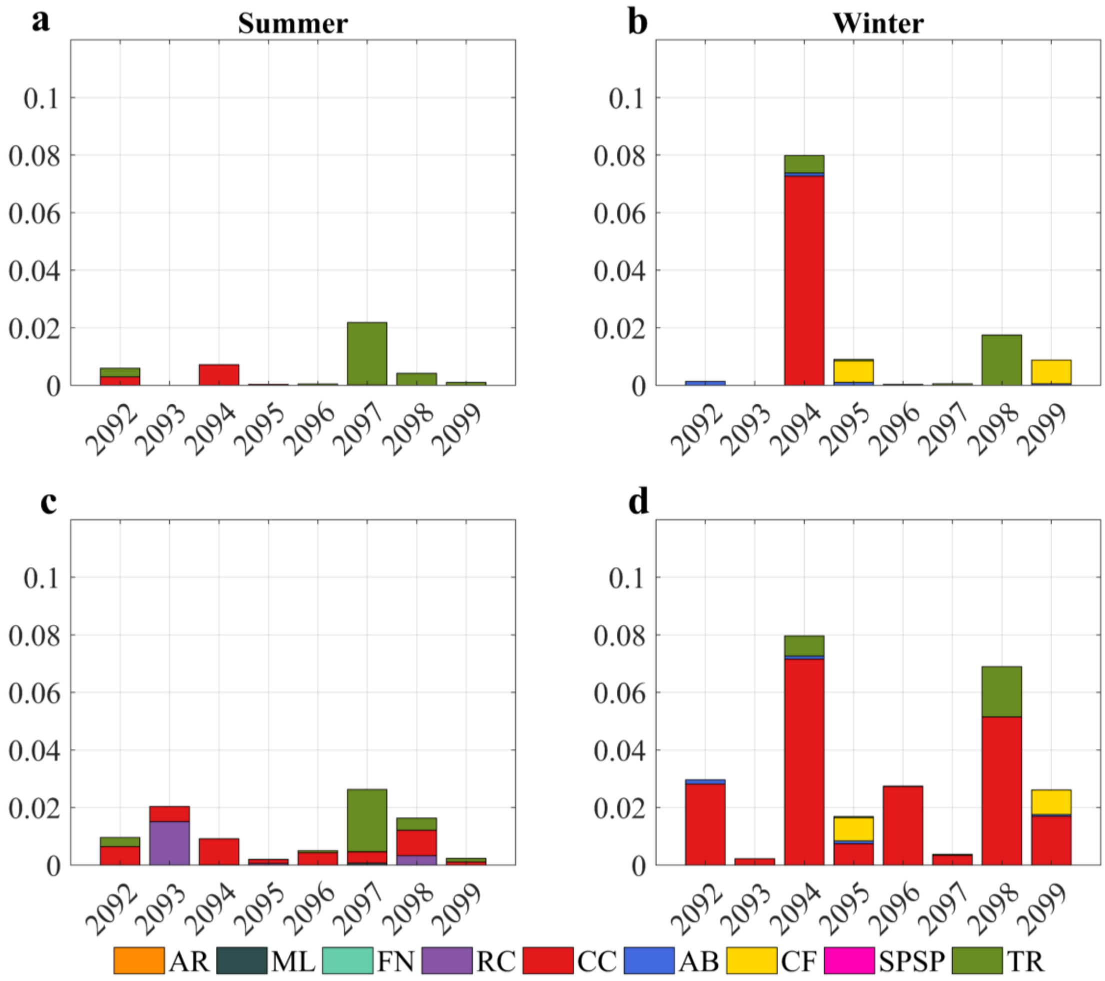

Supplementary Figure 11: Local retention per year as a proportion of total spawned particles in each MPA, considering the present-day thermal tolerance, in (a) summer and (b) winter; and considering acclimation of +3ºC in (c) summer and (d) winter. Each color represents the contribution per MPA in the total local retention per year. Created with Matlab R2019a (www.mathworks.com).

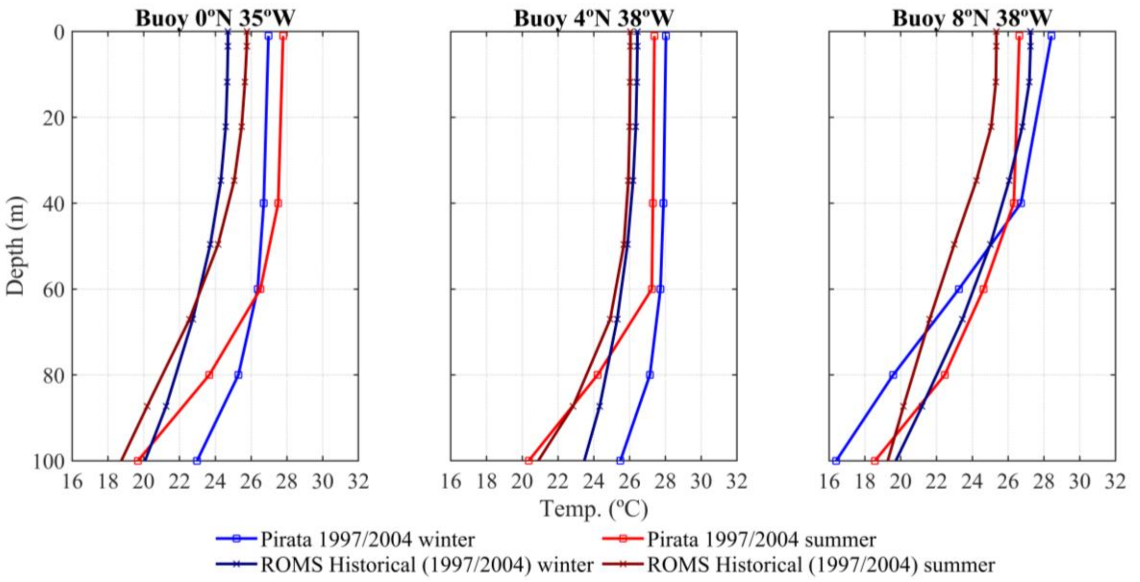

Supplementary Figure 12: Comparison between seasonal means of vertical temperature profiles from historical downscaling (1997 - 2004) and PIRATA buoys (1997 - 2004). Red tones represent summer profiles and blue tones represent winter profiles. Created with Matlab R2019a (www.mathworks.com).

Supplementary Table 1: List of ocean model atmospheric forcing variables and ocean boundary conditions derived from the HadGEM2-ES model, ensemble r2i1p1.

**Atmospheric components**	**Oceanic components**
huss: near-surface specific humidity	friver: water flux into sea water from rivers
pr: precipitation	so: sea water salinity
ps: surface air pressure	thetao: sea water potential temperature
lwrad: surface longwave radiation	uo: sea water X velocity
sward: surface shortwave radiation	vo: sea water Y velocity
tas: near-surface air temperature	zos: sea surface height above geoid
uas: Eastward near-surface wind	
vas: Northward near-surface wind	

Supplementary Table 2: Biological characteristics of *Sparisoma* and their respective values used in the ICHTHYOP (version 3.3) simulations.

**Variable**	**Value**
Quantity of particles	70000 particles [4]
Pelagic Larval Duration (PLD)	60 days [5]
Coastal behavior	Buouncing (larvae remains alive until the end of simulation)
Numerical scheme of advection	Runge-Kutta 4th Order [6]
Horizontal dispersion	10^{-9} $m^2.s^{-3}$ [7]
Release depth	20 meters[8]
Egg density	0.089 g. cm^3 [9]
Maximum egg age	24 hours [8]
Diel Vertical Migration (DVM) depth	58 m (day) and 27 m (night) [10]
Hour of sunrise and sunset	6:00 am and 18:00 pm
Actual temperature tolerance	24ºC to 30ºC [11–13]
Acclimation temperature tolerance	24º to 33ºC [14]

Supplementary Table 3: Description of simulation experiments and their respective time periods.

**Experiment**	**Period**
Historical Spin-up	1995 – 1996
Historical Experiment	1997 – 2005
RCP 8.5 Spin-up	2090 – 2091
RCP 8.5 Experiment	2092 – 2100
Summer Hourly RCP 8.5 Experiment	January – March (2092 – 2100)
Winter Hourly RCP 8.5 Experiment	July – September (2092 – 2100)

References

1. Talley, L. D., Pickard, G. L., Emery, W. J. & Swift, J. H. *Descriptive Physical Oceanography*. (Academic Press, 2011). doi:10.1016/C2009-0-24322-4.
2. Stramma, L. & England, M. On the water masses and mean circulation of the South Atlantic Ocean. *J. Geophys. Res. Ocean.* **104**, 20863–20883 (1999).

3. Peterson, R. G. & Stramma, L. Upper-level circulation in the South-Atlantic Ocean. *Prog. Oceanogr.* **26**, 1–73 (1991).
4. Endo, C. A. K., Gherardi, D. F. M., Pezzi, L. P. & Lima, L. N. Low connectivity compromises the conservation of reef fishes by marine protected areas in the tropical South Atlantic. *Sci. Rep.* **9**, 1–11 (2019).
5. Robertson, D. R., Karg, F., Leao de Moura, R., Victor, B. C. & Bernardi, G. Mechanisms of speciation and faunal enrichment in Atlantic parrotfishes. *Mol. Phylogenet. Evol.* **40**, 795–807 (2006).
6. Islam, M. A. A Comparative Study on Numerical Solutions of Initial Value Problems (IVP) for Ordinary Differential Equations (ODE) with Euler and Runge Kutta Methods. *Am. J. Comput. Math.* **05**, 393–404 (2015).
7. Peliz, A. *et al.* A study of crab larvae dispersal on the Western Iberian Shelf: Physical processes. *J. Mar. Syst.* **68**, 215–236 (2007).
8. Randall, J. E. & Randall, H. A. The spawning and early development of the Atlantic parrot fish, Sparisoma rubripinne, with notes on other scarid and labrid fishes. *Zoologica* **48**, 49–60 (1963).
9. Robertson, D. R. Egg size in relation to fertilization dynamics in free-spawning tropical reef fishes. *Oecologia* **108**, 95–104 (1996).
10. Huebert, K. B. Behavior and Transport of Pelagic Coral Reef Fish Larvae in the Straits of Florida. (UNIVERSITY OF MIAMI BEHAVIOR, 2009).
11. Bunn, N. A., Fox, C. J. & Webb, T. A literature review of studies on fish egg mortality: implications for the estimation of spawing stock biomass by the annual egg production method. *Sci. Ser. Tech. Rep.* **111**, 37 (2000).
12. Souza, C. S., Barreiro, A. S. & Mafalda Jr., P. O. Padrões espaciais e temporais de larvas de Scaridae (Pisces: Perciformes) no Nordeste do Brasil e suas relações com os fatores oceanográficos. *Braz. J. Aquat. Sci. Technol.* **14**, 1–11 (2010).
13. Van Rooij, J. M., Bruggemann, J. H., Videler, J. J. & Breeman, A. M. Ontogenetic, social, spatial and seasonal variations in condition of the reef herbivore Sparisoma viride. *Mar. Biol.* **123**, 269–275 (1995).
14. Donelson, J. M., Munday, P. L., Mccormick, M. I. & Nilsson, G. E. Acclimation to predicted ocean warming through developmental plasticity in a tropical reef fish. *Glob. Chang. Biol.* **17**, 1712–1719 (2011).
